# Fully Symmetric Cyclodextrin Polycarboxylates: How to Determine Reliable Protonation Constants from NMR Titration Data

**DOI:** 10.3390/ijms232214448

**Published:** 2022-11-21

**Authors:** Eszter Kalydi, Milo Malanga, Dóra Ujj, Gábor Benkovics, Zoltán Szakács, Szabolcs Béni

**Affiliations:** 1 Department of Pharmacognosy, Semmelweis University, Üllői út 26, H-1085 Budapest, Hungary; 2 CycloLab, Cyclodextrin R&D Ltd., Illatos út 7, H-1097 Budapest, Hungary; 3 Spectroscopic Research Department, Gedeon Richter Plc., H-1475 Budapest, Hungary

**Keywords:** cyclodextrins, sugammadex, ^1^H NMR pH-titration, protonation microconstant, cluster expansion model, parameter correlation

## Abstract

Acid-base properties of cyclodextrins (CDs), persubstituted at C-6 by 3-mercaptopropionic acid, sualphadex (Suα-CD), subetadex (Suβ-CD) and sugammadex (Suγ-CD, the antidote of neuromuscular blocking steroids) were studied by ^1^H NMR-pH titrations. For each CD, the severe overlap in protonation steps prevented the calculation of macroscopic p*K*_a_ values using the standard data fitting model. Considering the full symmetry of polycarboxylate structures, we reduced the number of unknown NMR parameters in the “Q-fitting” or the novel “equidistant macroscopic” evaluation approaches. These models already provided p*K*_a_ values, but some of them proved to be physically unrealistic, deceptively suggesting cooperativity in carboxylate protonations. The latter problem could be circumvented by adapting the microscopic site-binding (cluster expansion) model by Borkovec, which applies pairwise interactivity parameters to quantify the mutual basicity-decreasing effect of carboxylate protonations. Surprisingly, only a single averaged interactivity parameter could be calculated reliably besides the carboxylate ‘core’ microconstant for each CD derivative. The speciation of protonation isomers hence could not be resolved, but the optimized microscopic basicity parameters could be converted to the following sets of macroscopic p*K*_a_ values: 3.84, 4.35, 4.81, 5.31, 5.78, 6.28 for Suα-CD; 3.82, 4.31, 4.73, 5.18, 5.64, 6.06, 6.54 for Suβ-CD and 3.83, 4.28, 4.65, 5.03, 5.43, 5.81, 6.18, 6.64 for Suγ-CD. The pH-dependent charge of these compounds can now be accurately calculated, in support of designing new analytical methods to exploit their charge-dependent molecular recognition such as in cyclodextrin-aided chiral capillary electrophoresis.

## 1. Introduction

Cyclodextrins (CDs) are naturally occurring cyclic oligosaccharides, consisting of six, seven or eight glucopyranose units for α-, β- and γ-CD, respectively. CDs find a wide variety of applications in pharmaceutical sciences [1,2], cosmetic and food industries [3,4], environmental or analytical chemistry [5]. The molecular basis of these applications is the propensity of CDs to encapsulate small apolar organic molecules in their hydrophobic cavity [6,7,8], where the intrinsic chiral environment also enables enantiospecific molecular recognition, thus the use of CDs as chiral selectors in separation science [9]. In addition to their most common roles as solubilizer or stabilizer excipients for compounds of low aqueous solubility [10], some CDs also gained exquisite applications due to their highly specific interaction with small molecules [11].

**Figure 1:**
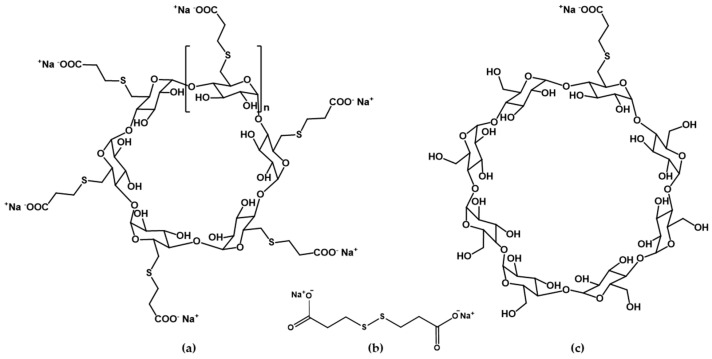
(**a**) Structures of sualphadex (Suα-CD, *n* = 1), subetadex (Suβ-CD, *n* = 2) and sugammadex (Suγ-CD, *n* = 3); (**b**) structure of 3,3′-dithiodipropionic acid (DTDPA); (**c**) structure of mono-sugammadex (mono-Suγ-CD).

Sugammadex (Suγ-CD) is a derivative of γ-CD, persubstituted by 3-mercaptopropionic acid sidechains on its primary hydroxyl groups (see Figure 1; Suγ-CD is in fact the octasodium salt of the polyacid). It is used in the clinical practice as the first selective antidote of steroidal neuromuscular blocking agents rocuronium and vecuronium during surgical interventions [12]. In addition, Suγ-CD was patented for the selective sequestration of cortisol [13] and fentanyl-related compounds [14] and applied as a selector in an affinity capillary electrophoresis method for penicillins [15] and cathinones [16]. The monosubstituted analogue of Suγ-CD, mono-Suγ-CD in Figure 1 has also been synthesized [17], without assessing its potential applications. In addition to the expanding utilization of Suγ-CD, there is also a growing interest in its α- and β-CD homologues sualphadex and subetadex [16,17], abbreviated as Suα-CD and Suβ-CD in Figure 1. Besides interaction studies with fentanyl [14], rocuronium bromide [18] and azobenzenes [19], their applicability as chiral selectors was also explored recently [16].

A thorough physicochemical profiling of CD derivatives is essential in order to deepen our understanding and improve the modeling perspectives on their intermolecular interactions upon inclusion complex formation. Although Suα-CD, Suβ-CD and Suγ-CD were first described twenty years ago [17], complete characterization of their acid-base properties has not yet been reported either experimentally or via computer prediction. The potentiometrically derived set of protonation constants of Suβ-CD published by Wenz et al. [20] is incomplete, giving only six of the seven values. There is also a recent example in the literature of p*K*_a_ determination for persubstituted CD-derivatives using ^13^C NMR spectroscopy, although the exploited method only provides a single average p*K*_a_ value for each binding site [21]. A full and reliable set of p*K*_a_ acid dissociation constants (or equivalently, stepwise log *K* protonation constants of their conjugate bases) enables the calculation of the net charge of these CD polycarboxylate anions for any arbitrary pH value. The ability to fine-tune the charge of the macromolecular host via solution pH and thus tailoring the selectivity of supramolecular recognition represent advantages of CDs with COOH-bearing side chains over their permanently charged sulfonated or sulfated counterparts [22]. Anionic CDs are used extensively as chiral selectors dissolved in the background electrolyte of capillary electrophoresis methods [23,24,25] or as mobile phase additives in chiral HPLC [5,26]. Persubstituted and monosubstituted CDs are so-called *single isomer* CDs, possessing well-defined molecular structures [25,27]. This feature enables the 3D structure of their complexes to be explored via NOE NMR studies or molecular modeling [28,29,30,31]. Thus, single isomer CDs offer a better experimental design and reproducibility for chiral separations, which is more difficult to attain with random substituted CD derivatives, with mixtures of CD isomers differing in degrees and positions of functionalization. These advantages generate an increasing demand for single isomer CDs in separation science.

The primary objective of the current study was to determine a reliable set of protonation constants for Suα-CD, Suβ-CD and Suγ-CD. The monobasic mono-Suγ-CD as well as the related bivalent small molecule, 3,3′-dithiodipropionic acid (DTDPA, Figure 1), modeling the isolated substituent side chain of the functionalized CDs were also characterized to investigate the impact of the local molecular environment on the intrinsic basicity of the carboxylate group(s).

The determination of protonation constants is now widely regarded as a routine task in physical chemistry, although published protonation data for at least hexabasic molecules are rare and often incomplete [32,33]. From the array of experimental methods available [34], potentiometric titration remains the gold standard technique for compounds available in sufficient amount and purity. Protonation constants have been successfully determined by pH-metric titrations for as large symmetric systems as dendrimers [35,36]. Another technique gaining immense popularity is NMR-pH titration, in which the chemical shift of the so-called *reporter nuclei* is followed as a function of solution pH [37,38]. Reporter nuclei are most often the carbon-bound protons located adjacent to the functional group undergoing protonation, but ^19^F, ^31^P, ^13^C or ^15^N NMR were also successfully applied [37,38,39,40]. The recorded sigmoidal (chemical shift vs. pH) titration curves undergo computer evaluation to extract the *macroscopic* log *K* or p*K*_a_ values [38,39,40,41]. Carefully selected NMR reporter nuclei enable even monitoring the protonation of each basic site individually. Site-specific or *microscopic protonation constants* (log *k* values) can be derived in favorable cases [37] and distribution curves can be computed for the *microspecies*, isomers holding the same number of protons, but differing in the site(s) of protonation [42]. Since the number of microspecies (2*^n^*) and microconstants (*n*2*^n^*^−1^) escalate rapidly with the growing number of binding sites (*n*), there is a principal limit for a complete, ab initio resolution of microequilibrium schemes of larger than triprotic molecules [43,44]. Molecular symmetry may reduce the number of independent basicity parameters significantly [43,45,46], since symmetry-equivalent groups possess identical basicities. Choosing one intrinsic microconstant for each site and group pair interactivity parameters as iteration parameters to build up all the remaining microconstants [45,46] represents a more efficient strategy for the computer fitting of NMR titration curves. The *site-binding (SB) or cluster expansion model* by Borkovec [47,48,49] succeeded in resolving the complete microspeciation of large symmetric polyprotic molecules such as linear polyamines or dendrimers.

Suα-CD, Suβ-CD and Suγ-CD CDs are *C_n_* symmetric polycarboxylate ligands. We applied ^1^H NMR-pH titrations for the acid-base profiling of these homo-perfunctionalized CDs. The strong overlap of six, seven or eight consecutive protonation steps resulted in single-sigmoid NMR titration curves, without any intermediate local plateaus. This latter feature aggravated the calculation of reliable protonation constants. Several computational strategies had to be explored to identify the most straightforward one in terms of precision and accuracy of the equilibrium constants.

## 2. Results

### 2.1. Acid-Base Characterization of Sualphadex

**Figure 2:**
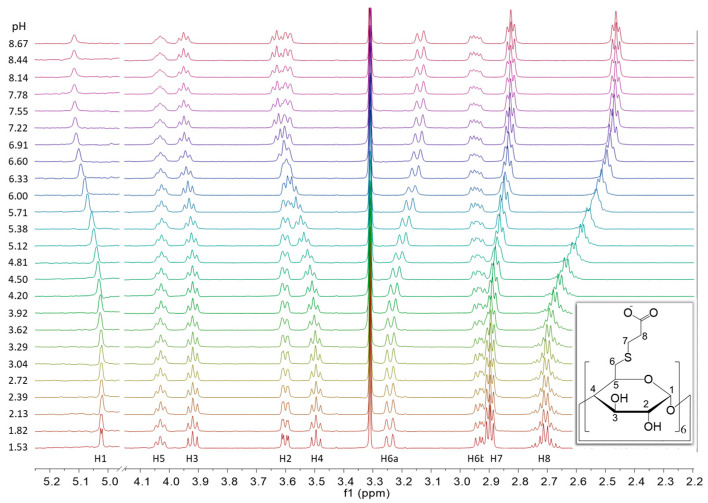
pH-dependent series of ^1^H NMR spectra of Suα-CD, with structure and atom numbering of the compound and assignment of the NMR signals (600 MHz, 298 K, H_2_O:D_2_O = 9:1).

**Figure 3:**
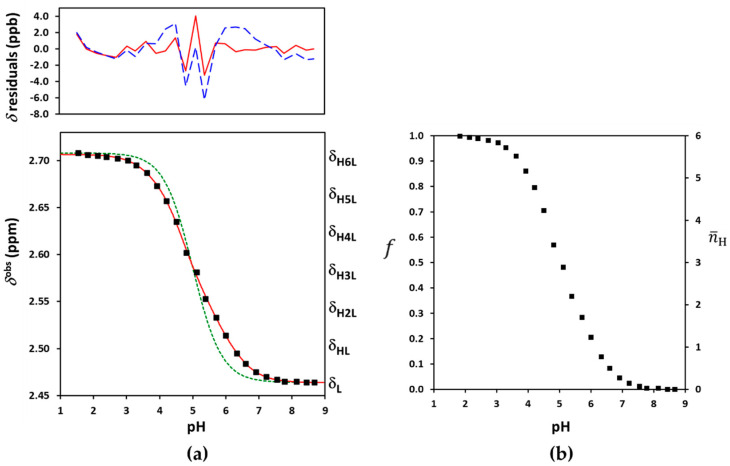
(**a**) The experimental NMR-pH titration curve of Suα-CD 8-CH_2_ protons in squares. The green dashed line is generated by fitting the site-binding model without p*ε*, while the solid red line represents the fits by the ED-macro, Q-fitting or the single-p$\bar{\varepsilon }$ site-binding models. The residuals by the ED-macro or the equivalent Q-fitting models are given in solid red line, while those by the single-p$\bar{\varepsilon }$ microscopic evaluation by dashed blue line above (see detailed description of these calculations in Section 2.3, Section 2.4 and Section 2.5); (**b**) protonation degree functions for individual carboxylates (*f*) and the whole Suα-CD molecule (${\bar{n}}_{H}$).

Figure 2 depicts the pH-dependent series of ^1^H NMR spectra of Suα-CD. From the signal shapes of the methine protons 1-CH through 5-CH, vicinal coupling constants can be derived, which can be in turn converted into dihedral angles and compared to the corresponding reference values of the native α-CD in water. Such an analysis may reveal the distortion of the CD macrocycle due to 6-persubstitution and its changes upon acid-base equilibria, but it may deserve a separate molecular modeling study. Returning to characterization of the acid-base equilibria, the 8-CH_2_ protons adjacent to the carboxylate group exhibit the largest change in chemical shift upon protonation, so these nuclei were selected as reporters. At slightly alkaline pH, their triplet signal is characteristic to the A_2_B_2_ spin system, which is gradually transformed into an AA’B_2_ type multiplet upon acidification. Regardless of the actual signal shape, the *center* of these multiplets can be precisely determined at each degree of titration and the resulting chemical shift values are plotted against pH in Figure 3a. This experimental ^1^H NMR titration curve was evaluated using the approaches detailed in the following sections.

### 2.2. The General Macroscopic Evaluation Model

**Figure 4:**
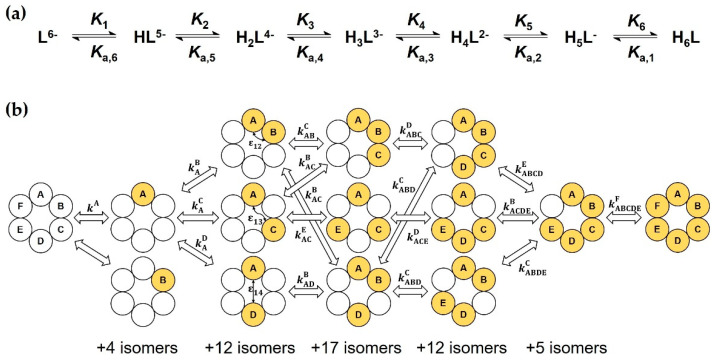
(**a**) Macroscopic protonation equilibria of Suα-CD, with protonation and dissociation macroconstants; (**b**) excerpt of the complete microscopic protonation scheme of Suα-CD. For each microspecies, empty circles symbolize the A, B, … F carboxylate sites in unprotonated form, while filled circles in protonated state. Microconstants $k_{y}^{x}$ and pair interactivity parameters $\varepsilon_{ij}$ are also indicated.

In the macroscopic description shown in Figure 4a, only the stoichiometry of the H*_i_*L species is considered, not their site(s) of protonation. Successive formation of the macrospecies (charges omitted) are characterized by the stepwise (log *K_i_*, p*K*_a,i_) or cumulative ($\log\;\beta_{i}$) macroconstants, whose relationships can be expressed as follows:

(1) $$\log\beta_{i}=\log\frac{\left[H_{i}L\right]}{\left[L\right]{\left[H^{+}\right]}^{i}}=\overset{i}{\underset{j=1}{\sum }}\log K_{j}=\overset{i}{\underset{j=1}{\sum }}pK_{a,n-j+1}$$

Owing to the rapid kinetics of proton exchange on the ^1^H NMR chemical shift timescale, the observed methylene peak position $\delta^{\mathrm{obs}}$ becomes a weighted average of the intrinsic chemical shifts $\delta_{H_{i}L}$ of the individual macrospecies [37]:

(2) $$\delta^{\mathrm{obs}}=\delta_{L}x_{L}+\delta_{\mathrm{HL}}x_{\mathrm{HL}}+\ldots +\delta_{H_{6}L}x_{H_{6}L}=\overset{n}{\underset{i=1}{\sum }}\delta_{H_{i}L}x_{H_{i}L}$$

The $x_{i}$ weighting factors are the pH-dependent molar fractions of the macrospecies, which can be expressed in terms of the $\beta_{i}$ formation constants as follows [32]:

(3) $$\delta_{\;\;}^{\mathrm{obs}}=\frac{\delta_{L}+\sum_{i=1}^{n}\delta_{H_{i}L}\beta_{i}{\left[H^{+}\right]}^{i}}{1+\sum_{i=1}^{n}\beta_{i}{\left[H^{+}\right]}^{i}}=\frac{\delta_{L}+\sum_{i=1}^{n}\delta_{H_{i}L}10^{\log\beta_{i}-i\;\mathrm{pH}}}{1+\sum_{i=1}^{n}10^{\log\beta_{i}-i\;\mathrm{pH}}}$$

In principle, the pH-independent limiting chemical shifts $\delta_{L}$ and $\delta_{H_{6}L}$ can be read off as alkaline and acidic plateaus of the titration curve in Figure 3a. In practice, these are co-iterated parameters with the log $\beta_{i}$ (or log *K_i_*) values. Due to absence of local plateaus, the five intermediates $\delta_{H_{i}L}$ values necessarily become iteration parameters. All the tested software tools including our own R scripts failed to estimate the seven $\delta_{H_{i}L}$ and the six log *K_i_* parameters together. The severe overlap of six protonation steps rendered the gold standard macroscopic evaluation unfeasible for the fully-symmetric Suα-CD. In our view, the culprit for this failure was the strong mathematical correlation between the $\delta_{H_{i}L}$ and log *K_i_* parameters. A similar conclusion emerged from the NMR titrations of complexones [46] and a two-step CD-steroid equilibrium system [50].

### 2.3. The Equidistant Macroscopic Evaluation Model

To avoid the correlation of spectroscopic and equilibrium parameters, the NMR titration curve of Suα-CD was interpreted from a different viewpoint. Due to the *C_n_* symmetry, each carboxylate group experiences the same f degree of protonation upon acidification: $f=f_{A}=f_{B}=\ldots =f_{F}$. As evident from Figure 3b, the f(pH) function ranges from 0 to 1 and it is linearly proportional to the observed displacement of the 8-methylene signal,

(4) $$\delta^{\mathrm{obs}}\left(\mathrm{pH}\right)=\delta_{L}+\Delta \delta \cdot f\left(\mathrm{pH}\right)$$

where $\Delta \delta =\delta_{H_{6}L}-\delta_{L}$ is the protonation increment [37]. On the other hand, a similar protonation degree function can be defined for the whole Suα-CD molecule:

(5) $${\bar{n}}_{H}\left(\mathrm{pH}\right)=\overset{n}{\underset{i=1}{\sum }}i\;x_{H_{i}L}\left(\mathrm{pH}\right)=\frac{\sum_{i=1}^{n}i\;10^{\log\beta_{i}-i\;\mathrm{pH}}}{1+\sum_{i=1}^{n}10^{\log\beta_{i}-i\;\mathrm{pH}}}$$

The ${\bar{n}}_{H}$ function was introduced by Bjerrum and plays a central role in the calculation of protonation constants from potentiometric titration curves. Figure 3b demonstrates that ${\bar{n}}_{H}$ ranges from 0 to *n* = 6, being the sum of contributions of the six equivalent carboxylates [43],

(6) $${\bar{n}}_{H}=f_{A}+f_{B}+\ldots +f_{F}=6f$$

Expressing f from Equation (4) and substituting it into Equation (6) yields the following formula:

(7) $${\bar{n}}_{H}\left(\mathrm{pH}\right)=6\frac{\delta^{obs}\left(\mathrm{pH}\right)}{\Delta \delta }-6\frac{\delta_{L}}{\Delta \delta }$$

This equation states that apart from the subtracted, pH-independent term, the experimental NMR titration curve $\delta^{obs}\left(\mathrm{pH}\right)$ is directly proportional to the ${\bar{n}}_{H}$ function. In other words, the evaluation of potentiometric and NMR titration curves becomes mathematically equivalent for a fully symmetric polyacid. Rearranging Equation (7) for $\delta^{obs}$ and combining it with Equation (5) yields the following NMR fitting function:

(8) $$\delta^{obs}=\delta_{L}+\Delta \delta \frac{\sum_{i=1}^{n}\frac{i}{n}10^{\log\beta_{i}-i\;\mathrm{pH}}}{1+\sum_{i=1}^{n}10^{\log\beta_{i}-i\;\mathrm{pH}}}=\frac{\delta_{L}+\sum_{i=1}^{n}\left(\delta_{L}+\frac{i\Delta \delta }{n}\right)10^{\log\beta_{i}-i\;\mathrm{pH}}}{1+\sum_{i=1}^{n}10^{\log\beta_{i}-i\;\mathrm{pH}}}$$

A comparison of Equation (8) with the general Equation (3) reveals that for fully symmetric ligands, the intermediate $\delta_{H_{i}L}$ values increase in strictly equidistant steps from $\delta_{L}$ to $\delta_{H_{6}L}=$ $\delta_{L}$ + Δδ (see Figure 3a). They cease to be iteration parameters, reducing the parameter space considerably.

This equidistant (ED) macroscopic evaluation already converged in both Microcal Origin and our R-script, yielding statistically sound values for the optimized parameters: $\delta_{L}$ = 2.4638 (standard deviation: 0.0008) ppm, $\delta_{H_{6}L}$ = 2.7064 (0.0008) ppm and the log *K* protonation constants collected in Table 1 (see Table S1 for more details). This approach succeeded in diminishing the correlation among equilibrium and NMR parameters, see Table S2. At the first sight, the resulting log *K* values seemed to be physically realistic. Nevertheless, a subtle problem with log *K*_5_ was traced by the monotonicity test of microconstants detailed in the following chapter. We note that potentiometric titration would also be prone to yield biased macroconstants for persubstituted CDs, since the fitting of model Equations (5) and (8) is equivalent from the mathematical statistical viewpoint.

### 2.4. The Q-Fitting Model

In the microscopic protonation scheme in Figure 4b, all protonation isomers of the H*_i_*L macrospecies (*i* = 1 to 5) are considered as distinct entities. The hexabasic Suα-CD ligand has 2^6^ = 64 protonation microspecies, whose formation are interconnected by 6 × 2^5^ = 192 microconstants $k_{y}^{x}$, whose *x* upper index identifies the protonating, the lower index *y* (if any) the already protonated carboxylate(s). Fortunately, molecular symmetry significantly reduces the number of constitutionally different microspecies. For instance, the following isomers protonated at two adjacent carboxylate groups are indistinguishable: AB ≡ BC ≡ CD ≡ DE ≡ EF ≡ FA. Thus, the multiplicity of microspecies AB becomes six. Considering all such symmetry-based simplifications, a reduced microscopic scheme with only 13 different microspecies can be constructed (see Figure S26) and certain microconstants coincide, e.g., $k^{A}=k^{B}$ or $k_{\mathrm{AC}}^{E}=k_{\mathrm{EA}}^{C}$, etc. A micro-formation constant denoted by κ [43,45] is defined for each microspecies, which reads, e.g., for microspecies ABD as follows:

(9) $$\kappa_{\mathrm{ABD}}=\frac{\left[\mathrm{ABD}\right]}{\left[L\right]{\left[H^{+}\right]}^{3}}=k^{A}k_{A}^{B}k_{\mathrm{AB}}^{D}=k^{A}k_{A}^{D}k_{\mathrm{AD}}^{B}\;\;\;\mathrm{etc}.$$

In contrast to the stepwise *k* microconstants, the 12 κ-values (with κ_L_ being 1 by definition) constitute a non-redundant set of parameters for the complete description of the protonation network [43].

The protonation degree function of carboxylate A is the concentration sum of all the microspecies protonated at this site, divided by the total ligand concentration,

(10) $$f_{A}=\frac{\left[A\right]+\left[\mathrm{AB}\right]+\left[\mathrm{AC}\right]+\ldots +\left[\mathrm{ABC}\right]+\left[\mathrm{ABD}\right]+\ldots +\left[\mathrm{ABCDEF}\right]}{\left[L\right]+\left[\mathrm{HL}\right]+[H_{2}L]+[H_{3}L]+[H_{4}L]+[H_{5}L]+[H_{6}L]}$$

Rewriting this equation in terms of equilibrium constants yields the following ratio of 6-degree polynomials,

(11) $$f_{A}\left(\mathrm{pH}\right)=\frac{Q_{1}\left[H^{+}\right]+Q_{2}{\left[H^{+}\right]}^{2}+Q_{3}{\left[H^{+}\right]}^{3}+Q_{4}{\left[H^{+}\right]}^{4}+Q_{5}{\left[H^{+}\right]}^{5}+Q_{6}{\left[H^{+}\right]}^{6}}{1+\beta_{1}\left[H^{+}\right]+\beta_{2}{\left[H^{+}\right]}^{2}+\beta_{3}{\left[H^{+}\right]}^{3}+\beta_{4}{\left[H^{+}\right]}^{4}+\beta_{5}{\left[H^{+}\right]}^{5}+\beta_{6}{\left[H^{+}\right]}^{6}}$$

where both the $Q_{i}$ and $\beta_{i}$ coefficients can be expressed as linear combinations of the 12 unknown $\kappa_{j}$ micro-formation constants:

(12) $$Q_{1}=\kappa_{A}=1\beta_{1}/6$$

(13) $$Q_{2}=2\kappa_{\mathrm{AB}}+2\kappa_{\mathrm{AC}}+\kappa_{\mathrm{AD}}=2\beta_{2}/6$$

(14) $$Q_{3}=3\kappa_{\mathrm{ABC}}+\kappa_{\mathrm{ACE}}+6\kappa_{\mathrm{ABD}}=3\beta_{3}/6$$

(15) $$Q_{4}=4\kappa_{\mathrm{ABCD}}+4\kappa_{\mathrm{ABCE}}+2\kappa_{\mathrm{ABDE}}=4\beta_{4}/6$$

(16) $$Q_{5}=5\kappa_{\mathrm{ABCDE}}=5\beta_{5}/6$$

(17) $$Q_{6}=6\kappa_{\mathrm{ABCDEF}}=6\beta_{6}/6$$

It was demonstrated earlier [43,44,45] that fitting of an Equation (11) type ratio of polynomials yields unequivocal results only for the $Q_{i}$ coefficients. Any attempt to compute the constituent $\kappa_{j}$ constants led to overparametrization of the regression for more than triprotic molecules. Fitting the combination of Equations (4) and (11) to the experimental dataset of Suα-CD yielded an excellent fit (Figure 3a) and statistically sound results for the iterated log $Q_{1-6}$, $\delta_{L}$ and Δδ parameters (see Tables S3 and S4). The log $Q_{1-6}$ coefficients were then used to back-calculate the log $\beta_{i}$ values by Equations (12)–(17), the differences of which yielded the stepwise log *K_i_* constants listed in Table 1. The resulting macroconstants, quality of fit and even the residual distributions coincide for the ED-macro and Q-fitting models, the two models are mathematically isomorphous.

The physical validity of the macroconstants was assessed by the monotonicity test of microconstants. For symmetric ligands with n fully equivalent basic sites, a set of stepwise $k_{i}$ microconstants can directly be derived from the macroconstants [37,43,51]:

(18) $$k_{i}=\frac{i}{n-i+1}K_{i}$$

The “first” and “last” microconstants calculated in this way coincide with the “true” microconstants of Suα-CD, $k_{1}=k^{A}$ and $k_{6}=k_{\mathrm{ABCDE}}^{F}$. However, each remaining intermediate $k_{i}$ value becomes an average of all microconstants describing the formation of protonation isomers of $H_{i}L$. For this reason, these averaged microconstants are denoted by ${\bar{k}}_{i}$ in Table 1. The majority of calculated ${\bar{k}}_{i}$ values decrease in a monotonic order as expected. However, coordination of the fifth proton to tetraprotonated microspecies seemingly increases the carboxylate basicity from log ${\bar{k}}_{4}$ = 4.82 to log ${\bar{k}}_{5}$ = 4.93. Such a positive cooperativity of the carboxylates would require a specific “allosteric” conformational transition of the CD to ease the subsequent protonation step. This type of allosterism is known for proton- or ligand-binding of certain proteins, but it is less conceivable for Suα-CD. In fact, the outlier nature of ${\bar{k}}_{5}$ has a root cause in the numerical sensitivity of the evaluation. Consequently, the macroconstants obtained by the ED-macro or Q-fitting approaches are still approximate values.

### 2.5. The Microscopic Site-Binding Model

Unbiased macroconstants for Suα-CD could only be deduced by the site-binding model (SB). Its mathematical elaboration has been published by Borkovec [47,48,49]; only the key concept is reiterated here using our notational system.

As a first approximation, the basicity of a given carboxylate is assumed to change negligibly upon successive protonation of the five others. This assumption seems to be plausible, considering the large covalent and spatial distances among the carboxylates. The formation constants of all conceivable protonation isomers of H*_i_*L simply become $\kappa_{i}={\left(k^{A}\right)}^{i}$ and $k^{A}$ can be termed as group constant in this statistical case [51]. Plugging these κ values into Equations (11)–(17) and using Equation (4) for nonlinear regression yields the results in Tables S5 and S6. However, the quality of fit in Figure 3a is clearly unsatisfactory, indicating that the six carboxylates of Suα-CD do not obey the statistical case of independent protonation.

As carboxylate interactions obviously cannot be neglected, all $k_{y\ldots }^{x}$ microconstants are expressed in terms of the ‘intrinsic’ or ‘core’ $k^{A}$ microconstant of the first protonating carboxylate and the three pair-interactivity parameters $\varepsilon_{12},\;\;\varepsilon_{13}$ and $\varepsilon_{14}$ (see Figure 4b). $\varepsilon_{12}$ quantifies how protonation of a carboxylate impacts the basicity at the adjacent site and vice versa. $\varepsilon_{13}$ and $\varepsilon_{14}$ describe the interaction of more distant sites. As already mentioned, negative cooperativity has been experimentally observed for the vast majority of small-molecules ranging from diacids to dendrimers [37,51], thus $\varepsilon_{\mathrm{AB}}$ < 1 or $p\varepsilon_{\mathrm{AB}}=-\log\varepsilon_{\mathrm{AB}}$ > 0. Taking the microspecies ABDE from Figure 4b as an example, its microconstant for the ABD→ABDE protonation step and its κ formation constant are expressed in terms of the site-binding model as follows:

(19) $$k_{\mathrm{ABD}}^{E}=k^{A}\varepsilon_{12}\varepsilon_{13}\varepsilon_{14}$$

(20) $$\kappa_{\mathrm{ABDE}}=k^{A}k_{A}^{D}k_{\mathrm{AD}}^{B}k_{\mathrm{ABD}}^{E}={\left(k^{A}\right)}^{4}\varepsilon_{12}^{2}\varepsilon_{13}^{2}\varepsilon_{14}^{2}$$

Similar κ values are formulated for the remaining twelve non-equivalent microspecies of Suα-CD. The $Q_{i}$ coefficients in Equations (11)–(17) also become functions of the four “cluster parameters” $k^{A}$, $\varepsilon_{12}$, $\varepsilon_{13}$ and $\varepsilon_{14}$. The master fitting Equation (4) brings two additional spectroscopic parameters ($\delta_{L}$ and Δδ) to this highly efficient parametrization.

The allowance for merely nearest-neighbor pair interactions via the parameter $\varepsilon_{12}$ yielded an excellent fit to the titration profile, see Figure 4a. The optimized log $k^{A}$ = 5.51 (0.02), p$\varepsilon_{12}$ = 0.44 (0.02), $\delta_{L}$ = 2.4650 (0.0009) ppm and Δδ = 0.241 (0.001) ppm parameters exhibited only moderate correlations (see Tables S7 and S8). For the sake of completeness, the calculation of two interactivity parameters (p$\varepsilon_{12}$ and p$\varepsilon_{13}$) was subsequently also attempted. The same excellent fit was accompanied here with satisfactory results only for three parameters: log $k^{A}$ = 5.63 (7), $\delta_{L}$ = 2.464 (0.001) ppm and Δδ = 0.241 (0.001) ppm. The optimized p$\varepsilon_{12}$ = 0.28 and p$\varepsilon_{13}$ = 0.28 parameters were burdened with disproportionately large standard deviations of 138 and a correlation coefficient of −1. The model turned out to be overparametrized with respect to the latter two interactivity parameters. This unexpected failure prompted us to perform extensive tests regarding the robustness of our SB fitting script. To this end, synthetic titration datasets were simulated from known cluster parameters and normally distributed random error added to the chemical shift values generated by Equation (3). We concluded that only one single pε value is accessible numerically in this symmetric hexaprotic scenario, any additional interactivity parameter becomes statistically insignificant. Moreover, the successfully optimized p$\varepsilon_{12}$ parameter did not exactly coincide with the p$\varepsilon_{12}$ value used for the simulation, rather it became a nontrivial combination of the input p$\varepsilon_{12}$, p$\varepsilon_{13}$ and p$\varepsilon_{14}$ values. Due to its averaged nature, the single accessible interactivity parameter will henceforth be denoted by p$\bar{\varepsilon }$. The inability to distinguish between 1 and 2, 1 and 3 or 1 and 4-type carboxylate interactions also implicates the distribution of protonation isomers which cannot be determined for a fully symmetric hexaprotic ligand. Nevertheless, the log $k^{A}$ = 5.51 and p$\bar{\varepsilon }$ = 0.44 results were combined to $\kappa_{i}$ via relationships similar to Equation (19), followed by their conversion to $\kappa_{i}$, $Q_{i}$, $\beta_{i}$, and finally to log $K_{i}$ equilibrium constants. The latter values listed in Table 1 represent the first reliable and physically sound protonation macroconstants for Suα-CD. They enable the calculation of speciation curves (in Figure S27) and the average charge of Suα-CD (in Figure S29) as a function of pH.

## 3. Discussion

### 3.1. Protonation of Subetadex and Sugammadex

**Figure 5:**
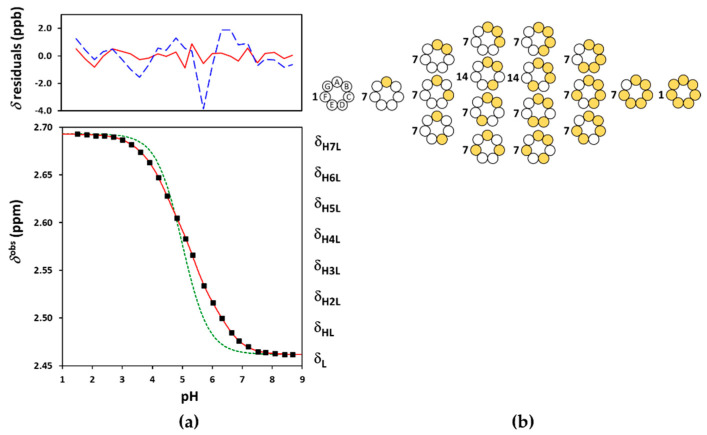
(**a**) The experimental NMR-pH titration curve of Suβ-CD 8-CH_2_ protons in squares. The green dashed line is generated by fitting the site-binding model without p*ε*, while the solid red line represents the fits by the ED-macro, Q-fitting or the single-p$\bar{\varepsilon }$ site-binding models. The residuals by the ED-macro or Q-fitting models given in solid red line, while those by the single-$p\bar{\varepsilon }$ microscopic evaluation by dashed blue line above; (**b**) the reduced microscopic protonation scheme of Suβ-CD, with multiplicities of the protonation isomers, where white circles indicate deprotonated-, and the yellow circles denote protonated binding sites.

**Figure 6:**
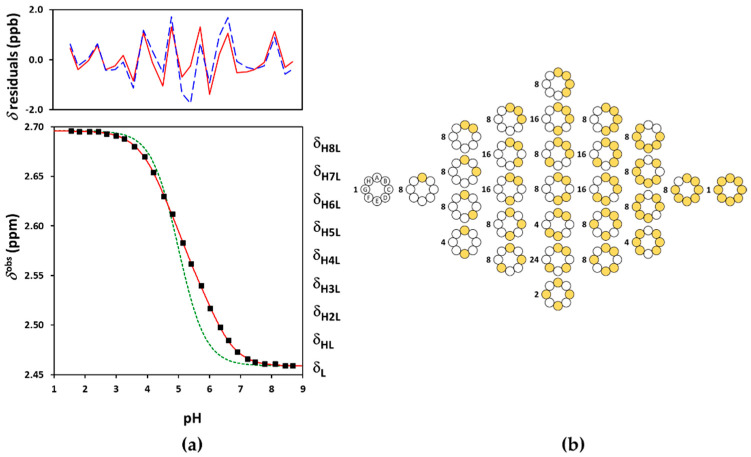
(**a**) The experimental NMR-pH titration curve of Suγ-CD 8-CH_2_ protons in squares. The green dashed line is generated by fitting the site-binding model without p*ε*, while the solid red line represents the fits by the ED-macro, Q-fitting or the single-p*ε* site-binding models. The residuals by the ED-macro or Q-fitting models given in solid red line, while those by the single-p$\bar{\varepsilon }$ microscopic evaluation by dashed blue line above; (**b**) the reduced microscopic protonation scheme of Suγ-CD, with multiplicities of the protonation isomers. The white circles indicate deprotonated-, and the yellow circles denote protonated binding sites.

The NMR-pH titration profiles and the problems of their evaluation were highly similar for the three investigated CD derivatives, so Suβ-CD and Suγ-CD are discussed together here. The macroscopic evaluation by Equation (3) failed for the larger CDs as well. The ED macroscopic and Q-fitting approaches yielded excellent fits (see Figure 5a and Figure 6a and Tables S9–S12 and S17–S20), but the resulting log *K* macroconstants were only partially meaningful. Table 1 reveals two incorrect log *K* values for Suβ-CD and one for Suγ-CD according to monotonicity test of the derived microconstants. We note that the macroconstant set published by Wenz et al. [20] also contains biased values. Henceforth we resorted to applying the site-binding model for Suβ-CD and Suγ-CD.

**Figure 7:**
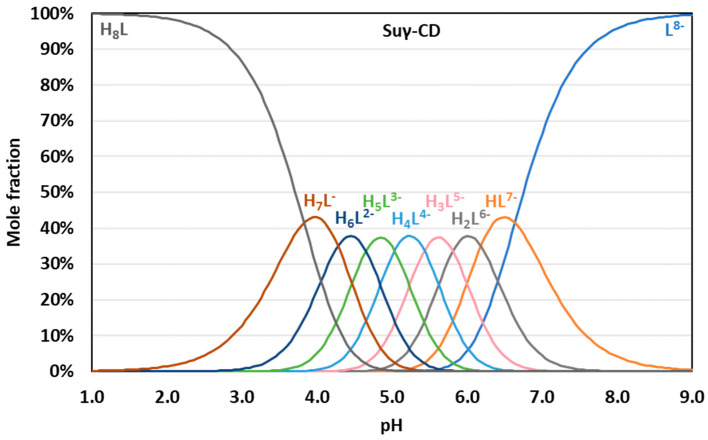
pH-dependent distribution of protonation microspecies of Suγ-CD.

Binding site combinatorics brings increasing complexity to the microscopic protonation schemes of the larger CDs: there are 128 microspecies (20 nonidentical when symmetry is considered, see Figure 5b) for Suβ-CD and 256 microspecies (36 nonidentical in the reduced protonation scheme in Figure 6b) for Suγ-CD. Assuming independent carboxylate protonations yielded a bad fit for both CDs, see the dashed lines in Figure 5a and Figure 6a. To allow for the pairwise basicity-modifying interactions of the carboxylates, p$\varepsilon_{12}$, p$\varepsilon_{13}$ and p$\varepsilon_{14}$ interactivity parameters were introduced for Suβ-CD, while an additional p$\varepsilon_{15}$ also emerged for of Suγ-CD. The excellent quality of solid-line fits displayed in Figure 5a and Figure 6a could be achieved with optimizing at least one pε value besides log *k*^A^ (Tables S13–S16 and S21–S24). Co-iteration of two or more pε values resulted in overparametrization also for these larger CDs, so merely an averaged p$\bar{\varepsilon }$ value could be extracted with statistical significance. The optimized parameters for Suβ-CD and Suγ-CD were converted to the stepwise log *K* macroconstants listed in Table 1. These latter values are more closely spaced, indicating less basicity-decreasing interactions between carboxylates when compared to the more steeply decreasing, biased values published by Wenz et al. [20]. The uniformity of p$\bar{\varepsilon }$ values ranging from 0.44 to 0.51 across the three Su-CD homologues corroborate the validity of our results. The macrospecies distribution curves for Suγ-CD are shown in Figure 7, while the same plot for Suβ-CD and the mean charge vs. pH profiles are given as Figures S28 and S29, respectively.

### 3.2. Basicity Comparison with Related Compounds

NMR-pH titrations were carried out also for the mono-Suγ-CD (Figures S30 and S31) and DTDPA model compounds (Figure S32). The standard macroscopic evaluation, Equation (3) for *n* = 1 and 2 yielded the protonation macroconstants without any difficulties, due to the significantly fewer iteration parameters. Table S25 lists the microscopic basicity parameters of the symmetric diacid DTDPA, calculated either from the macroconstants by known equations [37,45,51] or by any of the data fitting approaches discussed in Chapters 2.3–2.5. The log *K* = 4.35 (0.01) value of mono-Suγ-CD agrees well with the basicity of the first protonating carboxylate in DTDPA, log *k*^A^ = 4.33. The literature value for the latter constant is somewhat lower at 4.16 [52]. One would intuitively expect a similar intrinsic basicity for the thiopropionate side chain in the persubstituted CDs, since the carboxylates on different glucose units are seemingly isolated from one another (Figure 1). However, the log *k*^A^ values of 5.51, 5.70 (literature: 5.64 [20]) and 5.73 in Suα-CD, Suβ-CD and Suγ-CD reveal a consistently higher proton affinity for the same side chain. This surprising finding may be explained by the higher overall net charge (electron density) of the persubstituted CDs, and this phenomenon has also been revealed for acrylic acids where the p*K*_a_ of the monomeric form shows a 0.9 log *K* unit lower acidity compared to its polymeric form [53]. The averaged interactivity parameters of carboxylates, p$\bar{\varepsilon }$ ranging from 0.44 to 0.51 in the CDs are also higher than p*ε* = 0.15 in DTDPA. This latter value is reminiscent of carboxylate interactions (p*ε* ≈ 0.1) in flexible oligopeptides such as oxidized glutathione [45]. The covalently remote COO^−^ and COOH in monoprotonated DTDPA seem to approach each other by adopting a bent conformation. Earlier literature suggests almost independent carboxylate protonations with p*ε* ≈ 0 for DTDPA [51,52]. Turning back to the fully unprotonated CD polyanions, their side chains are expected to repel one another due to electrostatic reasons. However, this repulsion is presumably tamed upon successive protonation, so the side chain carboxylates on the pendant, flexible side chains may approach each other and even water-molecule-mediated hydrogen-bonding networks might be suspected at some stages of protonation. The “crowding” of COOH and COO^−^ groups near the narrower rim of the truncated cone of CD enhances basicity-modifying interactions of otherwise distant basic sites and this fact may be reflected in the experimentally found, seemingly elevated p$\bar{\varepsilon }$ > 0.4 values.

## 4. Materials and Methods

### 4.1. Materials

#### 4.1.1. Synthesis of Sugammadex-Analogues (General Procedure)

The per-6-iodo-CD was pre-solubilized in dimethyl sulfoxide (DMSO) in a Schott Duran bottle of adequate volume. In order to speed up the process of solubilization, the glass was sonicated at 50 °C. DMSO was added to the reaction vessel under inert atmosphere and 3-mercaptopropionic acid was then poured sequentially. The reaction mixture was cooled down in a water bath (*T* ~ 15 °C) and sodium methoxide-methanol solution was slowly added. The reaction mixture turned from a colorless solution to an intense pinkish solution with heat evolution. The DMSO-CD solution was added in one portion to the reaction mixture (Schott Duran is washed with 1 × 30 mL DMSO) causing almost immediate formation of a massive white precipitate. The suspension was stirred at r.t. for 1 h. The reaction mixture was filtered and the solid was extensively washed with methanol until a white solid was obtained. The solid was placed into a drying box and dried until constant weight.

Su-αCD: ^1^H NMR (400 MHz, 300 K, D_2_O) *δ*(ppm) 5.10 (d, *J* = 2.8 Hz, 6H, H1), 4.13 (bt, *J* = 8.5 Hz, 6H, H5), 3.99 (t, *J* = 9.2 Hz, 6H, H3), 3.67 (dd, *J* = 9.8, 2.8 Hz, 6H, H2), 3.58 (t, *J* = 8.8 Hz, 6H, H4), 3.29 (m, 6H, H6a), 2.98 (m, 18H, H6b, H7), 2.72 (m, 12H, H8).
^13^C NMR (100 MHz, 300 K, D_2_O) *δ*(ppm) 101.2 (C1), 84.6 (C4), 73.1 (C3), 71.8 (C2), 71.2 (C5), 35.4 (C8), 33.8 (C6), 28.3 (C7). The ^13^C chemical shifts were read from the DEPT-edited HSQC spectrum.
Su-βCD: ^1^H NMR (600 MHz, 300 K, D_2_O) *δ*(ppm) 5.18 (d, *J* = 3.5 Hz, 7H, H1), 4.14–3.85 (m, 14H, H3, H5), 3.77–3.51 (m, 14H, H2, H4), 3.33–3.10 (m, 7H, H6a), 3.12–2.75 (m, 18H, H6b, H7), 2.61 (t, *J* = 7.3 Hz, 14H, H8).
^13^C NMR (150 MHz, 300 K, D_2_O) *δ*(ppm) 178.5 (C9), 100.7 (C1), 83.1 (C4), 72.8 (C2), 71.9 (C3), 71.2 (C5), 36.6 (C8), 33.4 (C6), 28.8 (C7).
Su-γCD: ^1^H NMR (600 MHz, 300 K, D_2_O) *δ*(ppm) 5.22 (d, *J* = 3.8 Hz, 8H, H1), 4.010–4.02 (m, 8H, H5), 3.97 (d, *J* = 9.4 Hz, 8H, H3), 3.70 (dd, *J* = 9.8, 3.8 Hz, 8H, H2), 3.61 (t, *J* = 9.4 Hz, 8H, H4), 3.31–3.19 (m, 8H, H6a), 3.01 (dd, *J* = 13.5, 8.5 Hz, 8H, H6b), 2.97–2.87 (m, 16H, H7), 2.67 (t, *J* = 7.2 Hz, 16H, H8).
^13^C NMR (150 MHz, 298 K, D_2_O) *δ*(ppm) 178.4 (C9), 100.8 (C1), 82.7 (C4), 72.7 (C2), 72.2 (C3), 71.2 (C5), 36.1 (C8), 33.4 (C6), 28.5 (C7).

#### 4.1.2. Synthesis of Mono-Sugammadex

3-Mercaptopropionic acid (0.025 mol, 2.65 g, 2.2 mL) was added to a viscous DMSO (100 mL) solution of anhydrous 6-mono-*O*-(*p*-toluenesulfonyl)-γ-CD (14.06 g, 0.01 mol) under vigorous stirring and inert atmosphere. The yellowish reaction mixture was cooled down in water bath (T ~ 15 °C) and sodium methoxide-methanol solution (0.050 mol, 10.8 g, 11.4 mL) was slowly added. The reaction mixture turns from a yellowish solution to an intense pinkish solution under heat evolution. The solution was heated at 65 °C for 2 h and a white precipitate formed. The suspension was additionally heated at 65 °C for 3 h. The reaction mixture was cooled down, filtered and the solid was extensively washed with acetone (2 × 100 mL) and methanol (2 × 50 mL) until a white solid was obtained. The solid was placed into a drying box and dried until constant weight (7.5 g).

The structures of the synthetized compounds were confirmed using ESI-MS as well as routine 1- and 2-dimensional NMR techniques (see Figures S1–S25 in Supporting Information).

Mono-Su-γCD: ^1^H NMR (600 MHz, D_2_O, 300 K) *δ*(ppm) 5.22–5.04 (m, 8d, *J* = 3.8 Hz, 8H, H1, H1′), 4.08–3.99 (m, 1H, H5′), 3.99–3.80 (m, 29H, H3, H3′, H5, H5′, H6a,b), 3.72–3.63 (m, 8H, H2, H2′), 3.59 (m, 8H, H4, H4′), 3.14 (dd, *J* = 13.9, 2.5 Hz, 1H, H6′a), 2.93 (t, *J* = 7.1 Hz, 4H, H7*), 2.91–2.85 (m, 1H, H6′b), 2.83 (t, *J* = 7.4 Hz, 2H, H7′), 2.60 (t, *J* = 7.1 Hz, 4H, H8*), 2.48 (t, *J* = 7.4 Hz, 2H, H8′).
^13^C NMR (151 MHz, D_2_O) *δ*(ppm) 180.6 (C9′, C9*), 101.7–101.6 (C1), 101.4 (C1), 83.48 (C4′), 80.6–80.3 (C4), 72.9–72.7 (C3, C3′), 72.3 (C4), 71.7 (C5), 71.1 (C5′), 60.3–60.1 (C6), 37.48 (C8′), 36.76 (C8*), 34.58 (C7*), 32.85 (C6′), 29.01 (C7′).

The proton and carbon signals marked by * correspond to DTDPA, present as a byproduct formed during the synthesis and already described in the literature [54].

#### 4.1.3. Chemicals

D_2_O (99.96 atom % deuterium) and acetic acid-d_4_ (99.9 atom % deuterium) were purchased from Merck KGaA (Darmstadt, Germany). Methanol, sodium hydroxide (1 N solution), hydrochloric acid (1 N solution) and sodium chloride were reagent grade and purchased from Molar Chemicals Ltd. (Halásztelek, Hungary). Standard buffer solutions were purchased from VWR International LLC (Radnor, PA, USA). Water used for the solutions was obtained from a Milli-Q water purification system (Merck Millipore, Burlington, MA, USA).

### 4.2. Methods

#### 4.2.1. ESI-MS Measurements

Mass spectra were obtained on Bruker ESQUIRE 3000 ES-ion trap instrument with electrospray ionization (ESI) in negative mode. Samples were dissolved in water.

#### 4.2.2. pH Measurements

The pH meter readings were recorded using a Metrohm pH meter, equipped with a Metrohm 6.0234.110 combined glass electrode. A four-point calibration using pH 2.00, pH 4.00, pH 7.00 and pH 11.00 standard buffer solutions were performed directly before pH adjustments. The pH measurements were performed in 25 mL vessels with proper stirring before transferring 600 μL sample solutions into the NMR tubes. Solutions were prepared in the pH range of 1.50–8.70 with 0.30 increments using 0.9 M, 0.5 M and 0.1 M sodium hydroxide and hydrochloric acid solutions in H_2_O:D_2_O = 9:1.

#### 4.2.3. NMR Experiments

For structural characterizations of CD derivatives in D_2_O, a 600 MHz Varian DDR NMR spectrometer equipped with a 5 mm inverse-detection gradient probe or a 400 MHz Varian Mercury Plus spectrometer equipped with a 5mm Varian 400 Automation Triple Resonance Broadband Pulsed Field Gradient probe was used. For NMR-pH titrations, the same instruments were used. The pH-dependent series of ^1^H NMR spectra were recorded in a solvent mixture of H_2_O:D_2_O = 9:1 by volume, where the constant ionic strength and the buffer capacity were adjusted using 0.05 M sodium chloride and 0.05 M acetic acid-*d*_4_. An appropriate amount of CD was weighed to obtain a 1 mM solution and finally one drop of methanol was added as a chemical shift reference (3.310 ppm). The water resonance was diminished by the *dpfgse* pulse sequence [55] or presaturation. All the spectra were processed using the MestReNova v9.0.1-13254 (Mestralab Research, S.L., Santiago de Compostela, Spain) software.

#### 4.2.4. Evaluation of Titration Data

The standard macroscopic evaluation of NMR-pH titration curves (Equation (3) in Section 2.2) was performed with the following software tools: Microcal Origin Pro 8 (OriginLab Corp., Northampton, MA, USA), HypNMR2018 [38], OPIUM [39] and home-written R scripts (R version 4.1.2, The R Foundation, Vienna, Austria) using the *nls* module. The equidistant macroscopic (Equation (8)) and Q-fitting (Equations (4) and (11)) models were fitted both by Origin and our R-scripts to verify their equivalent performance regarding statistical description of the parameter estimates. The microscopic cluster expansion model in Section 2.5 requires summation of >100 variables which is unfeasible in the form of an explicit model function required by commercial data-fitting software like Origin. The enumeration of microspecies was therefore accomplished in a custom-written R script. To assess the robustness of our data-fitting model as well as the error propagation of the optimized log *k* and pε parameters to the derived log *K* macroconstants, Monte Carlo simulations [56,57] on >1000 replicates were performed in R.

## 5. Conclusions

Our study demonstrates that the full symmetry equivalence of basic sites in persubstituted CDs generates hitherto unrecognized difficulties when it comes to the determination of macroscopic protonation constants or p*K*_a_ values from NMR-pH titration curves. The standard macroscopic evaluation fails to converge, due to strong mathematical correlation between spectroscopic and equilibrium parameters. The Q-fitting or the novel equidistant macroscopic evaluation approaches reduce the dimensionality of parameter space and already yield macroconstant results. Due to symmetry, the latter values can be easily converted to microconstants, which are supposed to decrease monotonously upon proceeding protonation. This simple test is useful to discover systematic error in macroconstant values, thus it is recommended to use more extensively for polybasic compounds. On the other hand, the microscopic site-binding model of Borkovec produces microconstants and interactivity parameters as “building blocks” to guarantee the monotonicity of microconstants, thereby yielding an unbiased set of macroconstants. Surprisingly, we could demonstrate an inherent limitation of this approach in distinguishing group pair interactions according to their covalent distances, which is peculiar to fully-equivalent binding sites. While this problem hindered the resolution of the complete schemes of microscopic protonation equilibria, we publish here the first reliable sets of protonation macroconstants for the pharmacologically relevant sugammadex and its two homologues.

## Figures and Tables

**Table 1:** Macroscopic and averaged microscopic protonation constants of the studied cyclodextrin derivatives, with their standard deviations in parentheses. The ED macro and the Q-fitting evaluation approaches gave physically unrealistic values for some microconstants (suggesting cooperative proton uptake), the affected macro- and microconstants are marked by question marks. The site-binding microscopic evaluation yielded the correct protonation constants, whose errors were assessed by Monte Carlo simulations.

	Suα-CD		Suβ-CD			Suγ-CD	
	ED or Q	SB Micro	ED or Q	SB Micro	Lit. [20]	ED or Q	SB Micro
log *K*_1_	6.43 (0.07)	6.28 (0.02)	6.60 (0.03)	6.54 (0.02)	6.49 (1)	6.68 (0.08)	6.64 (0.02)
log *K*_2_	5.79 (0.09)	5.78 (0.02)	6.18 (0.04)	6.06 (0.02)	5.69 (1)	6.17 (0.12)	6.18 (0.02)
log *K*_3_	5.26 (0.11)	5.31 (0.02)	5.27 (0.08)	5.64 (0.02)	5.25 (1)	5.88 (0.16)	5.81 (0.02)
log *K*_4_	4.70 (0.14)	4.81 (0.02)	**5.53?** (0.06)	5.18 (0.01)	4.75 (1)	5.28 (0.22)	5.43 (0.01)
log *K*_5_	**4.53?** (0.11)	4.35 (0.02)	4.54 (0.06)	4.73 (0.02)	4.31 (1)	5.14 (0.22)	5.03 (0.01)
log *K*_6_	3.77 (0.07)	3.84 (0.02)	**4.46?** (0.05)	4.31 (0.02)	3.78 (1)	4.59 (0.17)	4.65 (0.02)
log *K*_7_	-	-	3.69 (0.03)	3.82 (0.02)	<3	**4.34?** (0.12)	4.28 (0.02)
log *K*_8_	-	-	-	-	-	3.78 (0.07)	3.83 (0.02)
$\log\;{\bar{k}}_{1}$	5.65 (0.07)	5.51 (0.02)	5.75 (0.03)	5.70 (0.02)	-	5.77 (0.08)	5.73 (0.02)
$\log\;{\bar{k}}_{2}$	5.39 (0.09)	5.38 (0.02)	5.71 (0.04)	5.58 (0.02)	-	5.63 (0.12)	5.64 (0.02)
$\log\;{\bar{k}}_{3}$	5.13 (0.12)	5.18 (0.02)	5.04 (0.08)	5.42 (0.02)	-	5.58 (0.16)	5.51 (0.02)
$\log\;{\bar{k}}_{4}$	4.83 (0.14)	4.94 (0.02)	5.53? (0.08)	5.18 (0.01)	-	5.18 (0.22)	5.33 (0.01)
$\log\;{\bar{k}}_{5}$	**4.93?** (0.11)	4.75 (0.02)	4.76 (0.06)	4.95 (0.02)	-	5.24 (0.22)	5.13 (0.01)
$\log\;{\bar{k}}_{6}$	4.55 (0.07)	4.62 (0.02)	**4.94?** (0.05)	4.78 (0.02)	-	4.89 (0.17)	4.95 (0.02)
$\log\;{\bar{k}}_{7}$	-	-	4.53 (0.03)	4.67 (0.02)	-	**4.89?** (0.12)	4.82 (0.02)
$\log\;{\bar{k}}_{8}$	-	-	-	-	-	4.69 (0.07)	4.73 (0.02)

## Data Availability

Not applicable.

## Supplementary Materials

The supporting information can be downloaded at: https://www.mdpi.com/article/10.3390/ijms232214448/s1.

## References

[1] Loftsson T., Jarho P., Másson M., Järvinen T. (2005). Cyclodextrins in drug delivery. Expert Opin. Drug Deliv..

[2] Challa R., Ahuja A., Ali J., Khar R.K. (2005). Cyclodextrins in drug delivery: An updated review. AAPS PharmSciTech.

[3] Matencio A., Navarro-Orcajada S., García-Carmona F., López-Nicolás J.M. (2020). Applications of cyclodextrins in food science. A review. Trends Food Sci. Technol..

[4] Fenyvesi É., Vikmon M., Szente L. (2016). Cyclodextrins in Food Technology and Human Nutrition: Benefits and Limitations. Crit. Rev. Food Sci. Nutr..

[5] Szente L., Szemán J. (2013). Cyclodextrins in analytical chemistry: Host-guest type molecular recognition. Anal. Chem..

[6] Saokham P., Muankaew C., Jansook P., Loftsson T. (2018). Solubility of Cyclodextrins and Drug/Cyclodextrin Complexes. Molecules.

[7] Pinho E., Grootveld M., Soares G., Henriques M. (2014). Cyclodextrins as encapsulation agents for plant bioactive compounds. Carbohydr. Polym..

[8] Capelezzo A.P., Mohr L.C., Dalcanton F., de Mello J.M.M., Fiori M.A. (2018). β-Cyclodextrins as Encapsulating Agents of Essential Oils. Cyclodextrin—A Versatile Ingredient.

[9] Schneiderman E., Stalcup A.M. (2000). Cyclodextrins: A versatile tool in separation science. J. Chromatogr. B Biomed. Sci. Appl..

[10] Garibyan A., Delyagina E., Agafonov M., Khodov I., Terekhova I. (2022). Effect of pH, temperature and native cyclodextrins on aqueous solubility of baricitinib. J. Mol. Liq..

[11] Várnai B., Grabarics M., Szakács Z., Pagel K., Malanga M., Sohajda T., Béni S. (2021). Structural characterization of fondaparinux interaction with per-6-amino-beta-cyclodextrin: An NMR and MS study. J. Pharm. Biomed. Anal..

[12] Singh D., Sivashanmugam T., Kumar H., Nag K., Parthasarathy S., Shetti A. (2013). Sugammadex: A revolutionary drug in neuromuscular pharmacology. Anesth. Essays Res..

[13] Zhang M., Hill D.R., Rees D. (2002). Use of Cortisol-Sequestering Agents for the Treatment of Hypercortisolaemia Related Disorders. WO Patent.

[14] Kennedy D.J., Mayer B.P., Valdez C.A. (2019). Modified Cyclodextrins for the Selective Sequestration of Fentanyl Related Compounds and Uses Thereof. U.S. Patent.

[15] Darwish K.A., Mrestani Y., Neubert R.H.H. (2013). Study of Interactions Between Sugammadex and Penicillins Using Affinity Capillary Electrophoresis. Chromatographia.

[16] Ujj D., Kalydi E., Malanga M., Varga E., Sohajda T., Béni S., Benkovics G. (2022). Sugammadex analogue cyclodextrins as chiral selectors for enantioseparation of cathinone derivatives by capillary electrophoresis. J. Chromatogr. A.

[17] Adam J.M., Bennett D.J., Bom A., Clark J.K., Feilden H., Hutchinson E.J., Palin R., Prosser A., Rees D.C., Rosair G.M. (2002). Cyclodextrin-Derived Host Molecules as Reversal Agents for the Neuromuscular Blocker Rocuronium Bromide: Synthesis and Structure−Activity Relationships. J. Med. Chem..

[18] Cameron K.S., Fielding L. (2002). NMR diffusion coefficient study of steroid-cyclodextrin inclusion complexes. Magn. Reson. Chem..

[19] Möller N., Hellwig T., Stricker L., Engel S., Fallnich C., Ravoo B.J. (2017). Near-infrared photoswitching of cyclodextrin–guest complexes using lanthanide-doped LiYF 4 upconversion nanoparticles. Chem. Commun..

[20] Wenz G., Strassnig C., Thiele C., Engelke A., Morgenstern B., Hegetschweiler K. (2008). Recognition of Ionic Guests by Ionic β-Cyclodextrin Derivatives. Chem. A Eur. J..

[21] Agnes M., Thanassoulas A., Stavropoulos P., Nounesis G., Miliotis G., Miriagou V., Athanasiou E., Benkovics G., Malanga M., Yannakopoulou K. (2017). Designed positively charged cyclodextrin hosts with enhanced binding of penicillins as carriers for the delivery of antibiotics: The case of oxacillin. Int. J. Pharm..

[22] Benkovics G., Fejős I., Darcsi A., Varga E., Malanga M., Fenyvesi É., Sohajda T., Szente L., Béni S., Szemán J. (2016). Single-isomer carboxymethyl-γ-cyclodextrin as chiral resolving agent for capillary electrophoresis. J. Chromatogr. A.

[23] Řezanka P., Navrátilová K., Řezanka M., Král V., Sýkora D. (2014). Application of cyclodextrins in chiral capillary electrophoresis. Electrophoresis.

[24] Yu R.B., Quirino J.P. (2019). Chiral Selectors in Capillary Electrophoresis: Trends During 2017–2018. Molecules.

[25] Fejős I., Kalydi E., Malanga M., Benkovics G., Béni S. (2020). Single isomer cyclodextrins as chiral selectors in capillary electrophoresis. J. Chromatogr. A.

[26] Rocco A., Maruška A., Fanali S. (2012). Cyclodextrins as a chiral mobile phase additive in nano-liquid chromatography: Comparison of reversed-phase silica monolithic and particulate capillary columns. Anal. Bioanal. Chem..

[27] Fejős I., Kalydi E., Kukk E.L., Seggio M., Malanga M., Béni S. (2021). Single Isomer N-Heterocyclic Cyclodextrin Derivatives as Chiral Selectors in Capillary Electrophoresis. Molecules.

[28] Servais A.-C., Rousseau A., Fillet M., Lomsadze K., Salgado A., Crommen J., Chankvetadze B. (2010). Separation of propranolol enantiomers by CE using sulfated β-CD derivatives in aqueous and non-aqueous electrolytes: Comparative CE and NMR study. Electrophoresis.

[29] Salgado A., Chankvetadze B. (2016). Applications of nuclear magnetic resonance spectroscopy for the understanding of enantiomer separation mechanisms in capillary electrophoresis. J. Chromatogr. A.

[30] Várnai B., Malanga M., Sohajda T., Béni S. (2022). Molecular interactions in remdesivir-cyclodextrin systems. J. Pharm. Biomed. Anal..

[31] Várnai B., Zsila F., Szakács Z., Garádi Z., Malanga M., Béni S. (2022). Sulfobutylation of Beta-Cyclodextrin Enhances the Complex Formation with Mitragynine: An NMR and Chiroptical Study. Int. J. Mol. Sci..

[32] Mirzahosseini A., Orgován G., Tóth G., Hosztafi S., Noszál B. (2015). The complete microspeciation of ovothiol A disulfide: A hexabasic symmetric biomolecule. J. Pharm. Biomed. Anal..

[33] Zhang H., Xue H., Yang J., Liang L. (2015). Determination of complex 12-grade phytic acid dissociation constants. Bulg. Chem. Commun..

[34] Reijenga J., van Hoof A., van Loon A., Teunissen B. (2013). Development of Methods for the Determination of pK a Values. Anal. Chem. Insights.

[35] Cakara D., Kleimann J., Borkovec M. (2003). Microscopic Protonation Equilibria of Poly(amidoamine) Dendrimers from Macroscopic Titrations. Macromolecules.

[36] van Duijvenbode R.C., Rajanayagam A., Koper G.J.M., Baars M.W.P.L., de Waal B.F.M., Meijer E.W., Borkovec M. (2000). Synthesis and Protonation Behavior of Carboxylate-Functionalized Poly(propyleneimine) Dendrimers. Macromolecules.

[37] Szakács Z., Kraszni M., Noszál B. (2004). Determination of microscopic acid-base parameters from NMR-pH titrations. Anal. Bioanal. Chem..

[38] Hägele G., Szakács Z., Ollig J., Hermens S., Pfaff C. (2000). NMR-controlled titrations: Characterizing aminophosphonates and related structures. Heteroat. Chem..

[39] Frassineti C., Ghelli S., Gans P., Sabatini A., Moruzzi M.S., Vacca A. (1995). Nuclear Magnetic Resonance as a Tool for Determining Protonation Constants of Natural Polyprotic Bases in Solution. Anal. Biochem..

[40] Vacca A., Ghelli S., Frassineti C., Alderighi L., Gans P., Sabatini A. (2003). Determination of protonation constants of some fluorinated polyamines by means of ^13^C NMR data processed by the new computer program HypNMR2000. Protonation sequence in polyamines. Anal. Bioanal. Chem..

[41] Kyvala M., Lukes I. OPIUM computer program. https://web.natur.cuni.cz/~kyvala/opium.html.

[42] Mazák K., Noszál B. (2016). Advances in microspeciation of drugs and biomolecules: Species-specific concentrations, acid-base properties and related parameters. J. Pharm. Biomed. Anal..

[43] Szakács Z., Noszál B. (1999). Protonation microequilibrium treatment of polybasic compounds with any possible symmetry. J. Math. Chem..

[44] Ullmann G.M. (2003). Relations between Protonation Constants and Titration Curves in Polyprotic Acids: A Critical View. J. Phys. Chem. B.

[45] Noszál B., Szakács Z. (2003). Microscopic Protonation Equilibria of Oxidized Glutathione. J. Phys. Chem. B.

[46] Szakács Z., Béni S., Noszál B. (2008). Resolution of carboxylate protonation microequilibria of NTA, EDTA and related complexones. Talanta.

[47] Borkovec M., Koper G.J.M. (2000). A Cluster Expansion Method for the Complete Resolution of Microscopic Ionization Equilibria from NMR Titrations. Anal. Chem..

[48] Borkovec M., Brynda M., Koper G.J.M., Spiess B. (2002). Resolution of microscopic protonation mechanisms in polyprotic molecules. Chimia.

[49] Borkovec M., Koper G.J.M., Spiess B. (2014). The intrinsic view of ionization equilibria of polyprotic molecules. New J. Chem..

[50] Al-Soufi W., Cabrer P.R., Jover A., Budal R.M., Tato J.V. (2003). Determination of second-order association constants by global analysis of ^1^H and ^13^C NMR chemical shifts. Steroids.

[51] Noszal B. (1986). Group constant: A measure of submolecular basicity. J. Phys. Chem..

[52] Hawkins C.J., Perrin D.D. (1963). Polynuclear Complex Formation. II. Copper(II) with Cystine and Related Ligands. Inorg. Chem..

[53] Xu L., Kamon Y., Hashidzume A. (2021). Synthesis of a New Polyanion Possessing Dense 1,2,3-Triazole Backbone. Polymers.

[54] Li X.-B., Li Z.-J., Gao Y.-J., Meng Q.-Y., Yu S., Weiss R.G., Tung C.-H., Wu L.-Z. (2014). Mechanistic Insights into the Interface-Directed Transformation of Thiols into Disulfides and Molecular Hydrogen by Visible-Light Irradiation of Quantum Dots. Angew. Chemie Int. Ed..

[55] Hwang T.L., Shaka A.J. (1995). Water Suppression That Works. Excitation Sculpting Using Arbitrary Wave-Forms and Pulsed-Field Gradients. J. Magn. Reson. Ser. A.

[56] Hu W., Xie J., Chau H.W., Si B.C. (2015). Evaluation of parameter uncertainties in nonlinear regression using Microsoft Excel Spreadsheet. Environ. Syst. Res..

[57] Madurga S., Nedyalkova M., Mas F., Garcés J.L. (2017). Ionization and Conformational Equilibria of Citric Acid: Delocalized Proton Binding in Solution. J. Phys. Chem. A.

