# Supplementary material for: Fully Symmetric Cyclodextrin Polycarboxylates: How to Determine Reliable Protonation Constants from NMR Titration Data
Source: Int J Mol Sci. 2022 Nov 21;23(22):14448. doi: 10.3390/ijms232214448 (PMC9696085; doi:10.3390/ijms232214448)
Supplement: Supplementary file 1 [file ijms-23-14448-s001.zip › ijms-2029871-supplementary.pdf]

International Journal of Molecular Sciences

Supplementary Material

Fully symmetric cyclodextrin polycarboxylates: how to determine reliable protonation constants from NMR titration data

Eszter Kalydi [1], Milo Malanga [2], Dóra Ujj [2], Gábor Benkovics [2], Zoltán Szakács [2] and Szabolcs Béni [1,*]

[1] Department of Pharmacognosy, Semmelweis University, Üllői út 26, H-1085 Budapest, Hungary
[2] Last affiliation: CycloLab, Cyclodextrin R&D Ltd., Illatos út 7, H-1097 Budapest, Hungary
[3] Spectroscopic Research Department, Gedeon Richter Plc., H-1475 Budapest, Hungary
* Correspondence: beniszabi@gmail.com or beni.szabolcs@pharma.semmelweis-univ.hu Tel.: +36 1 317 2979

Table of Contents

Ch1. Spectroscopic characterization of the CD derivatives 2

Ch2. The reduced microscopic protonation scheme of Sualphadex 14

Ch3. Detailed results of titration curve evaluations by R scripts 15

Ch4. Calculated macrospecies distributions and charge-pH profiles 21

Ch5. Acid-base profiling of related compounds 22

International Journal of *Molecular Sciences*

Ch1. Spectroscopic characterization of the CD derivatives

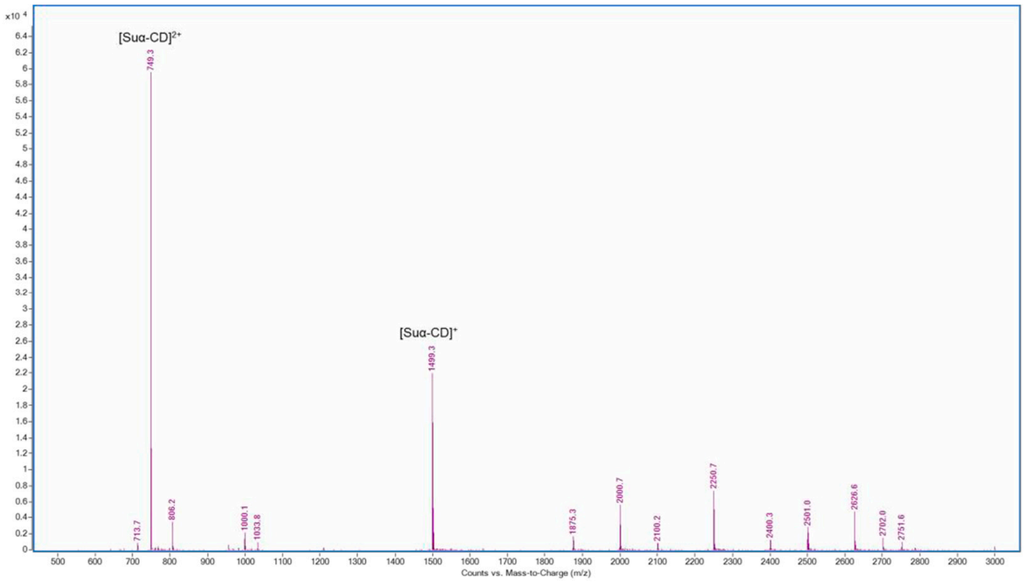

Figure S1. ESI-MS spectrum of Suα-CD.

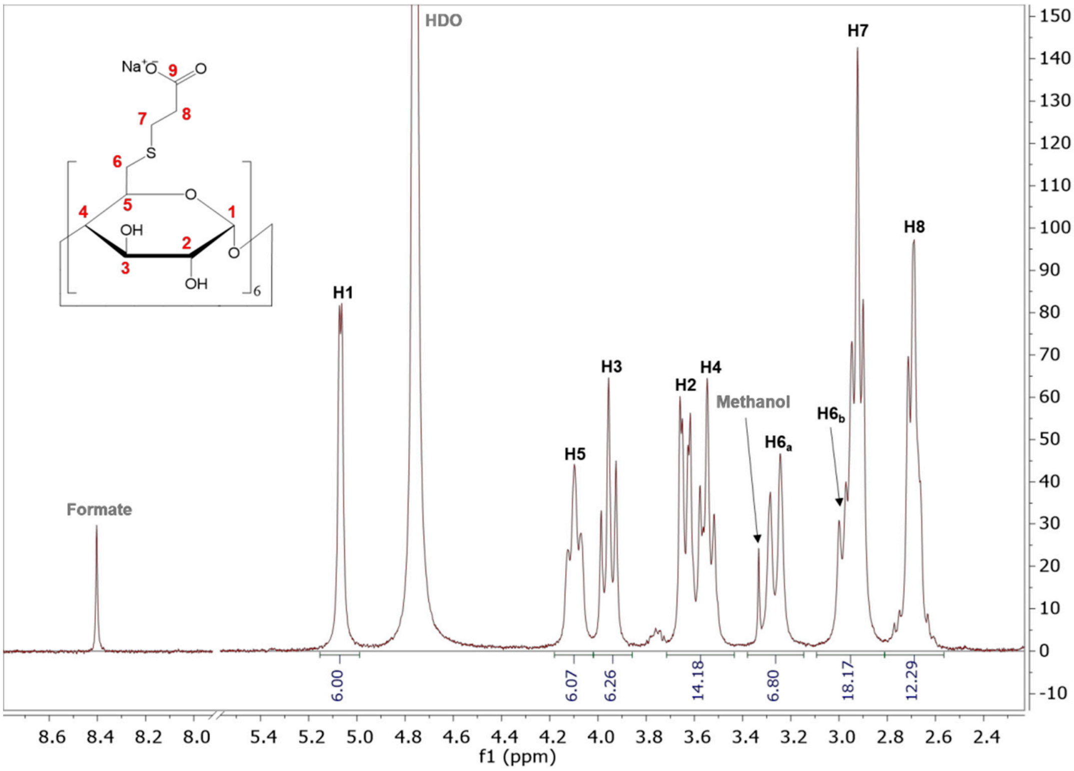

Figure S2. ^{1}H NMR spectrum of Suα-CD with full signal assignment (400 MHz, D_2O, 300 K).

International Journal of *Molecular Sciences*

MDPI

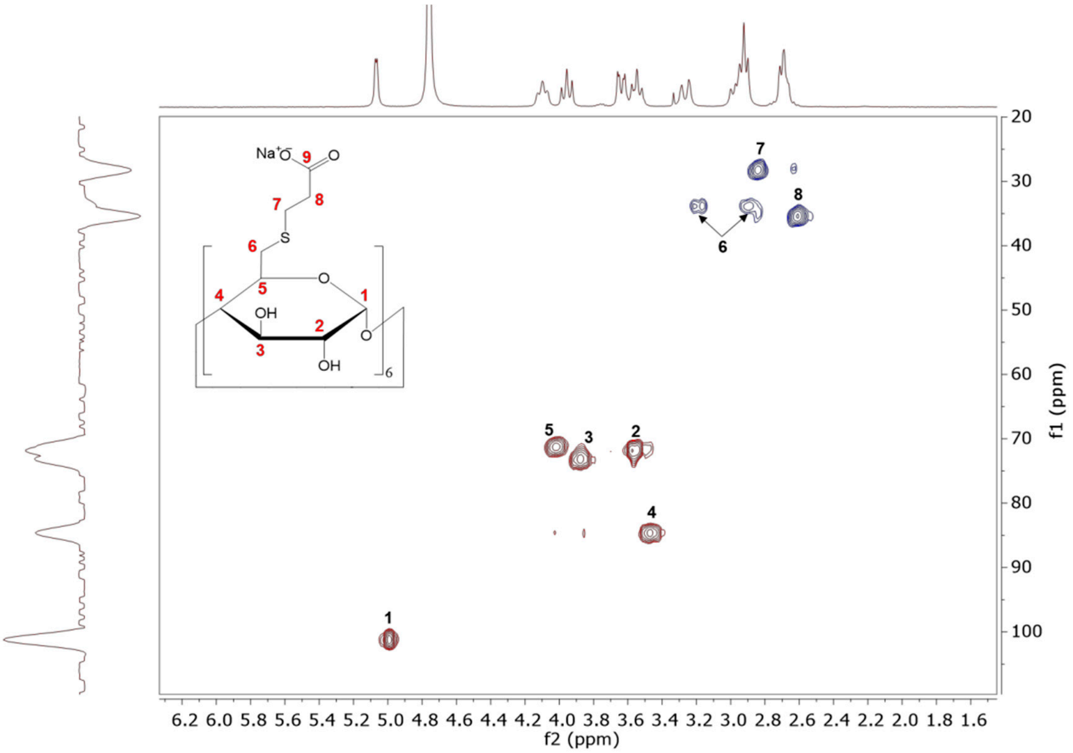

Figure S3. Multiplicity-edited HSQC spectrum of Suα-CD with full assignment (400 MHz, D_2O, 300 K).

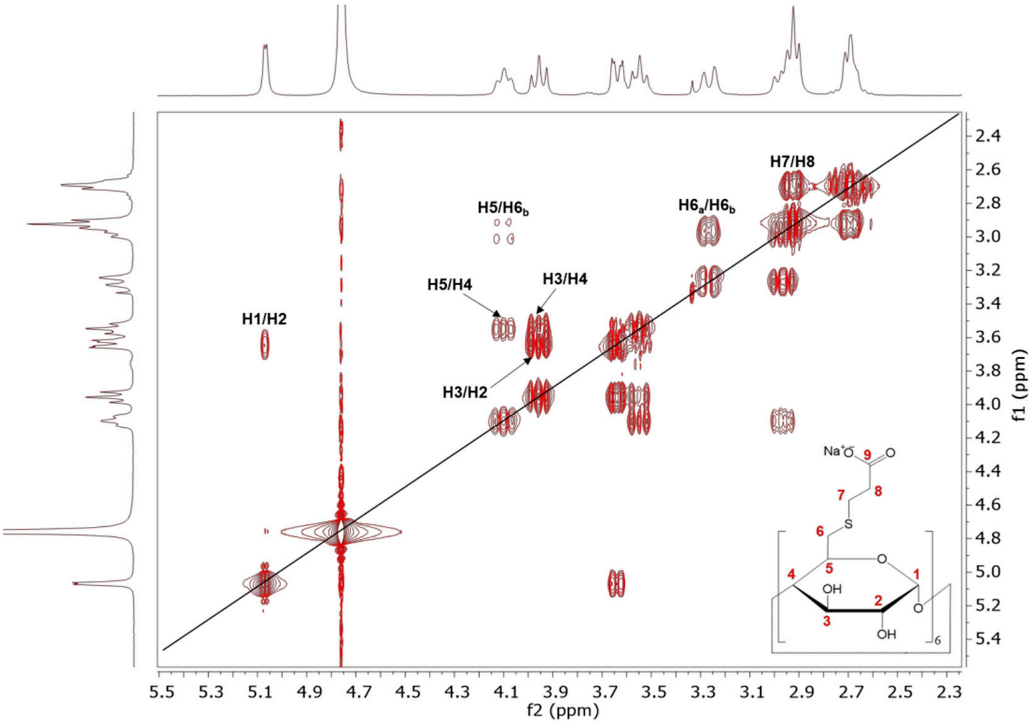

Figure S4. ^1H-^1H COSY spectrum of Suα-CD with full assignment (400 MHz, D_2O, 300 K).

International Journal of
Molecular Sciences

MDPI

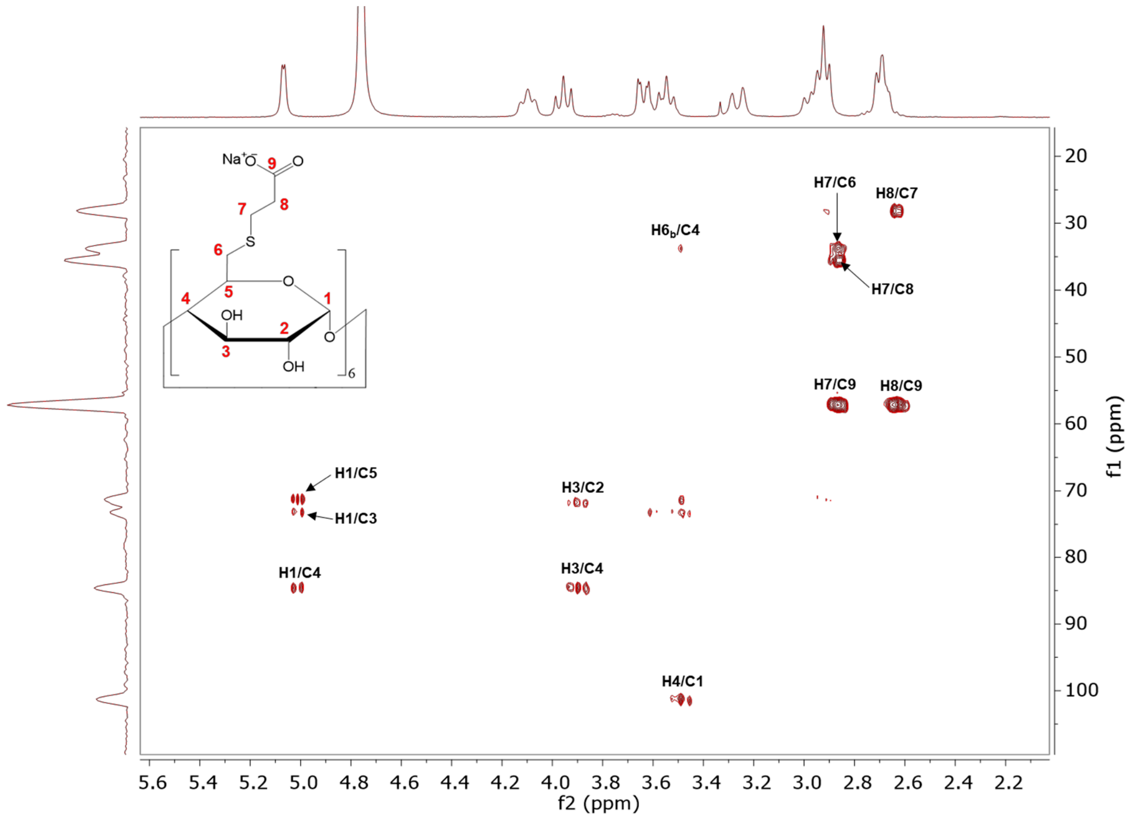

Figure S5. HMBC spectrum of Suα-CD with full assignment (400 MHz, D_2O, 300 K).

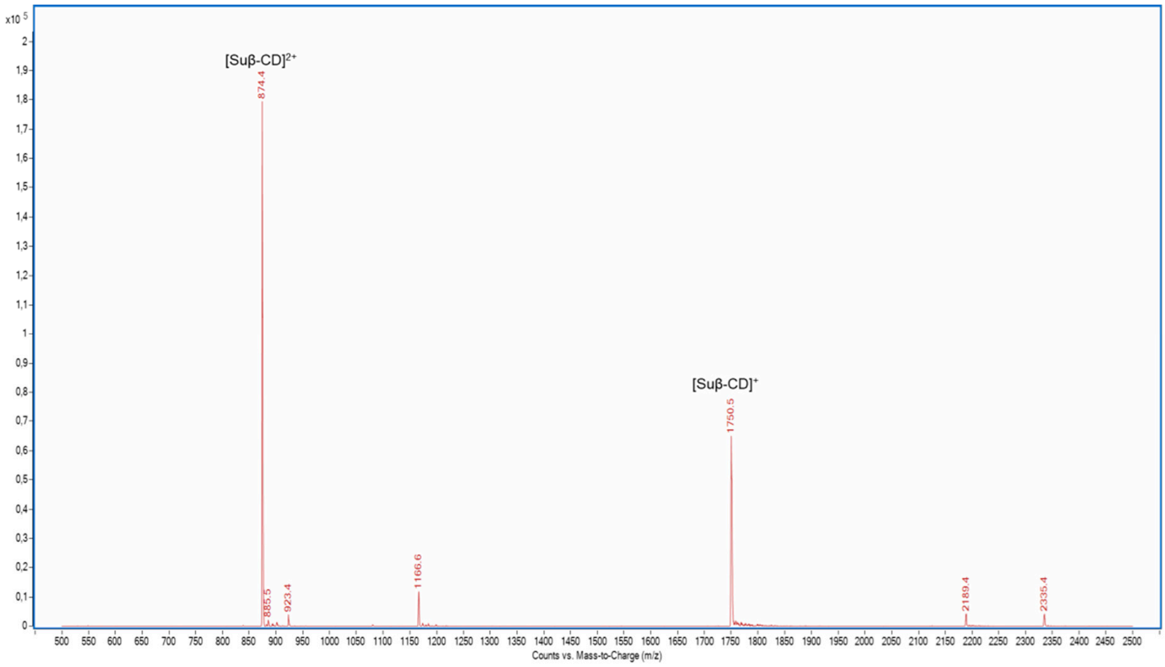

Figure S6. ESI-MS spectrum of Suβ-CD.

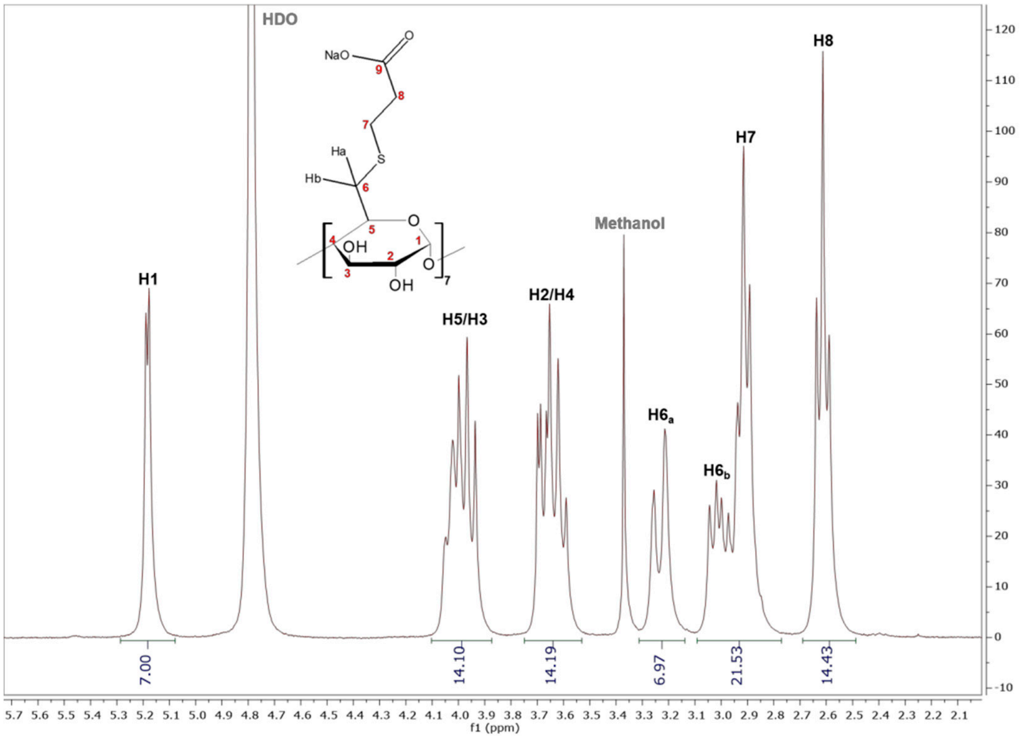

Figure S7. ^{1}H NMR spectrum of Suβ-CD with full assignment (600 MHz, D_2O, 300 K).

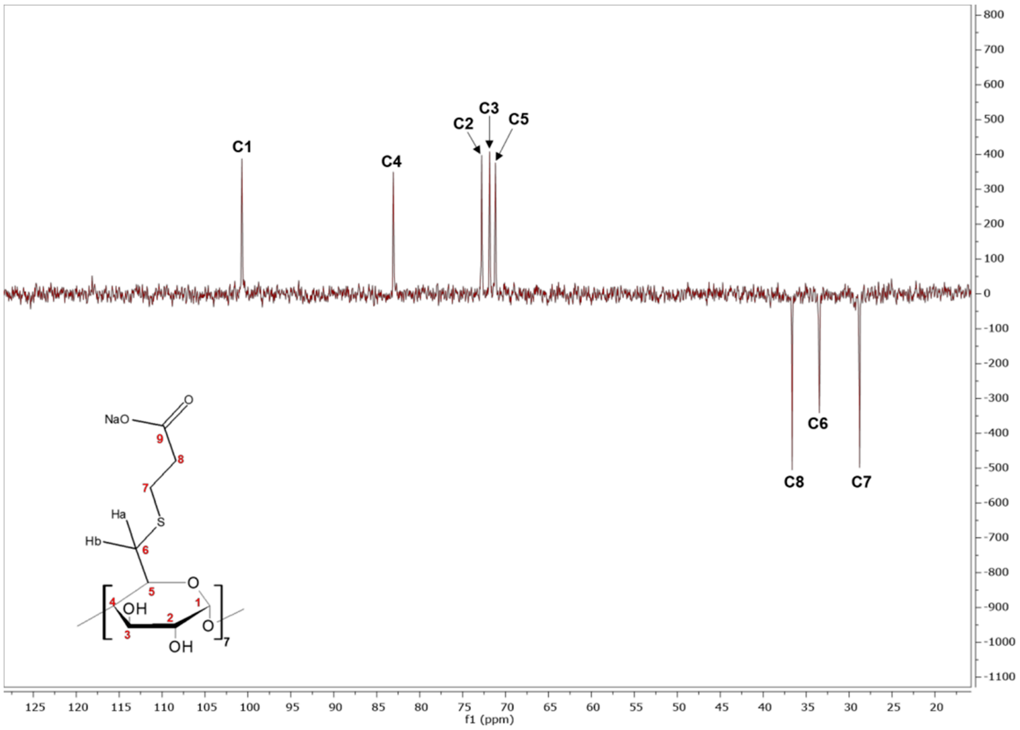

Figure S8. DEPT spectrum of Suβ-CD with full assignment (150 MHz, D_2O, 300 K).

International Journal of *Molecular Sciences*

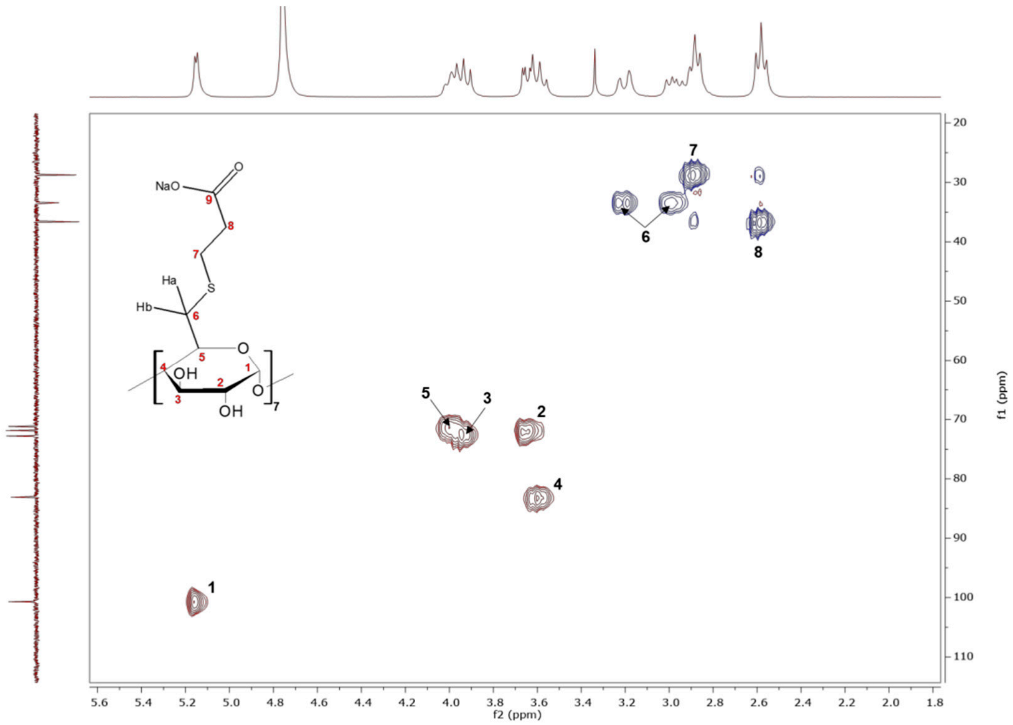

Figure S9. Multiplicity-edited HSQC spectrum of Suβ-CD with full assignment (600 MHz, D_2O, 300 K).

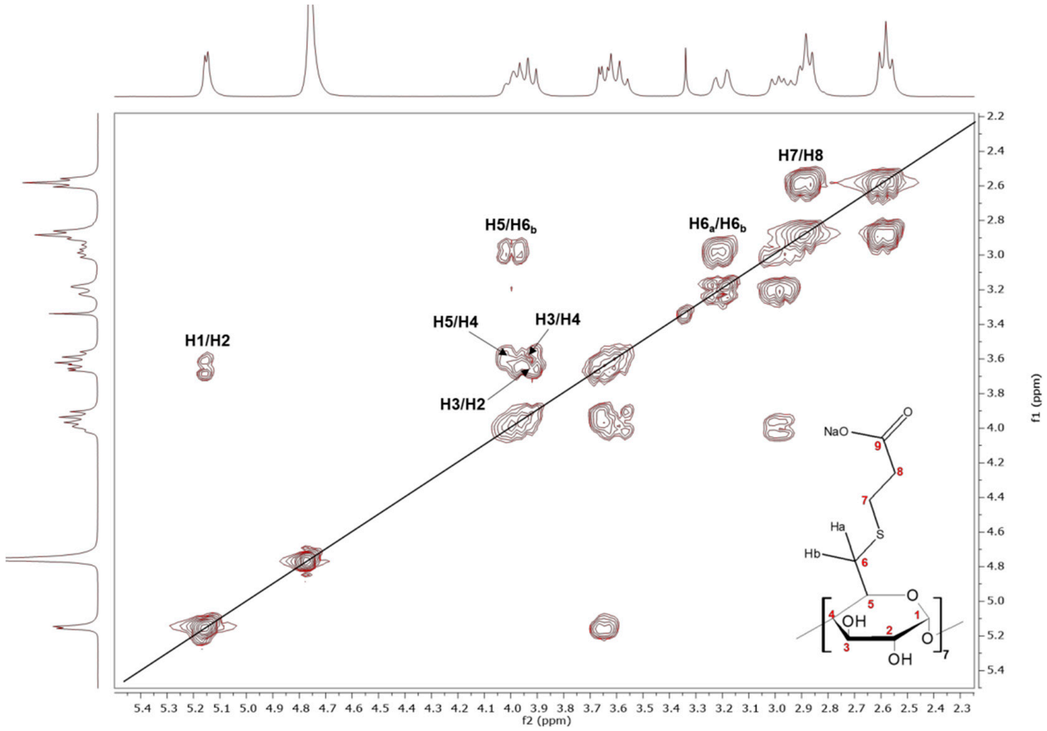

Figure S10. ^1H-^1H COSY spectrum of Suβ-CD with full assignment (600 MHz, D_2O, 300 K).

International Journal of *Molecular Sciences*

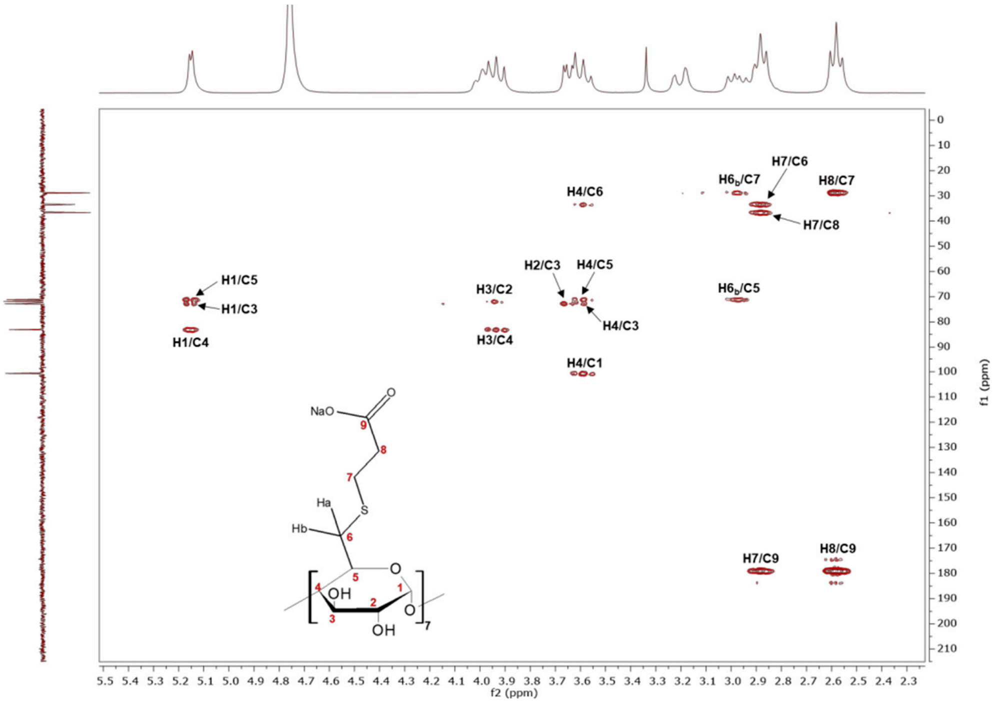

Figure S11. HMBC spectrum of Suβ-CD with full assignment (600 MHz, D_2O, 300 K).

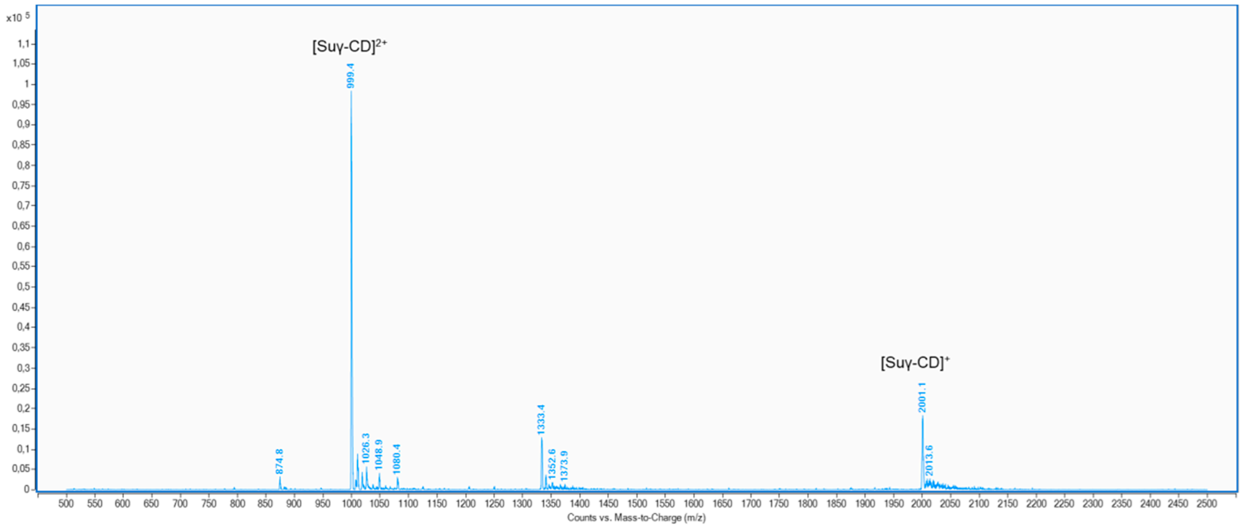

Figure S12. ESI-MS spectrum of Suγ-CD.

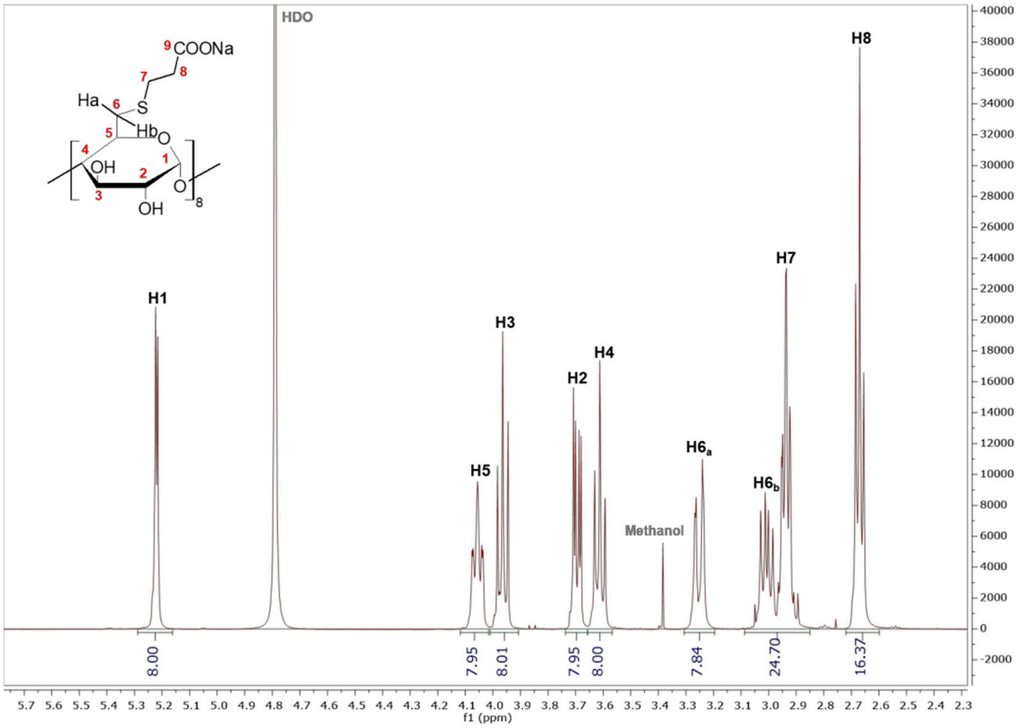

Figure S13. ^{1}H NMR spectrum of Suγ-CD with full assignment (600 MHz, D_2O, 300 K).

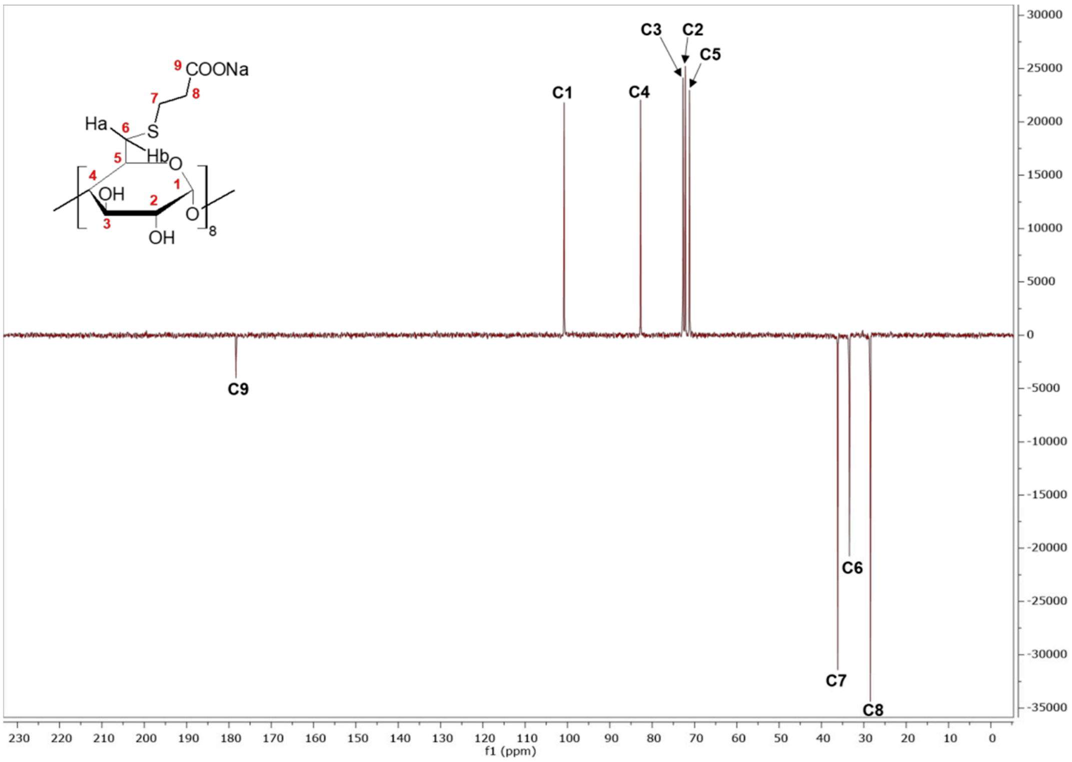

Figure S14. DEPT spectrum of Suγ-CD with full assignment (150 MHz, D_2O, 300 K).

International Journal of *Molecular Sciences*

MDPI

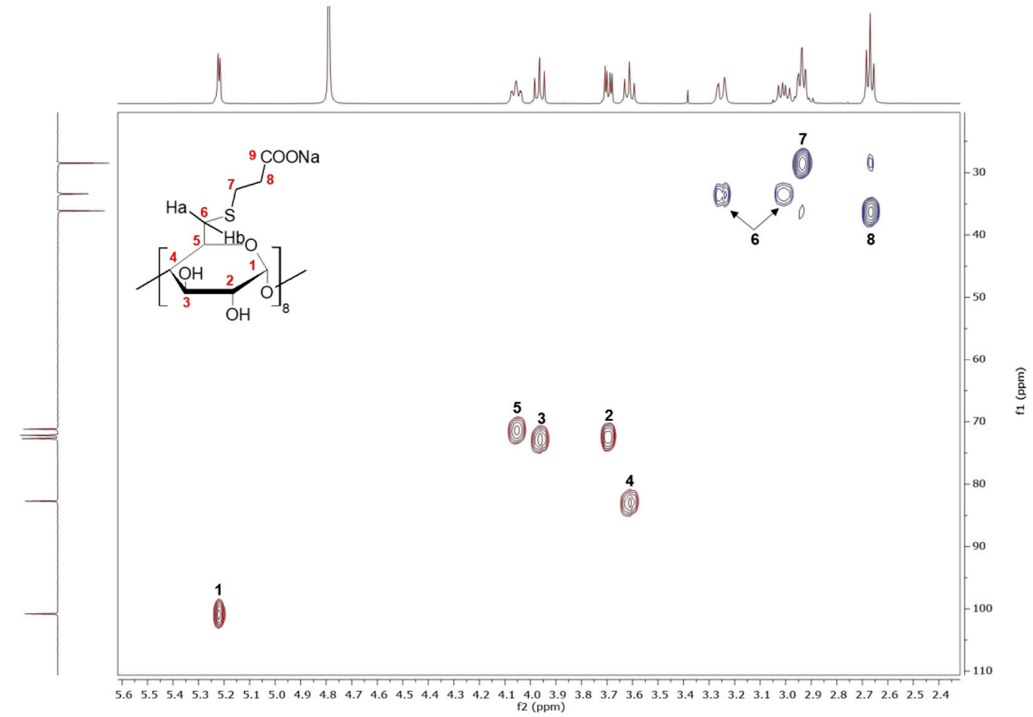

Figure S15. Multiplicity-edited HSQC spectrum of Suγ-CD with full assignment (600 MHz, D_2O, 300 K).

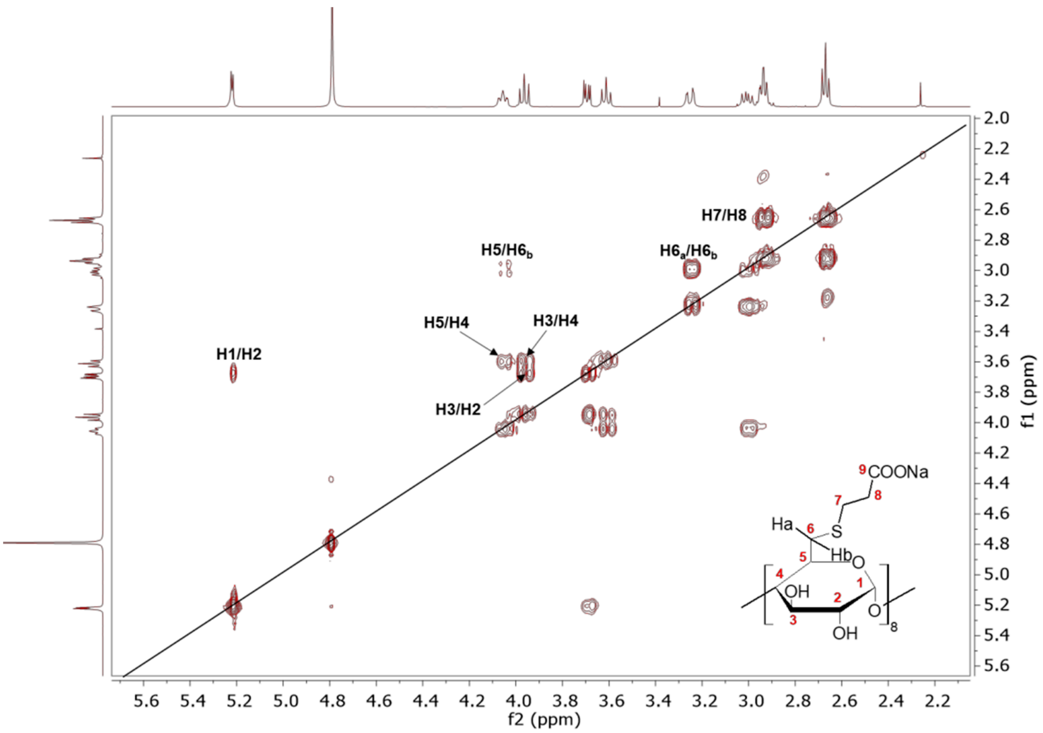

Figure S16. ^1H-^1H COSY spectrum of Suγ-CD with full assignment (600 MHz, D_2O, 300 K).

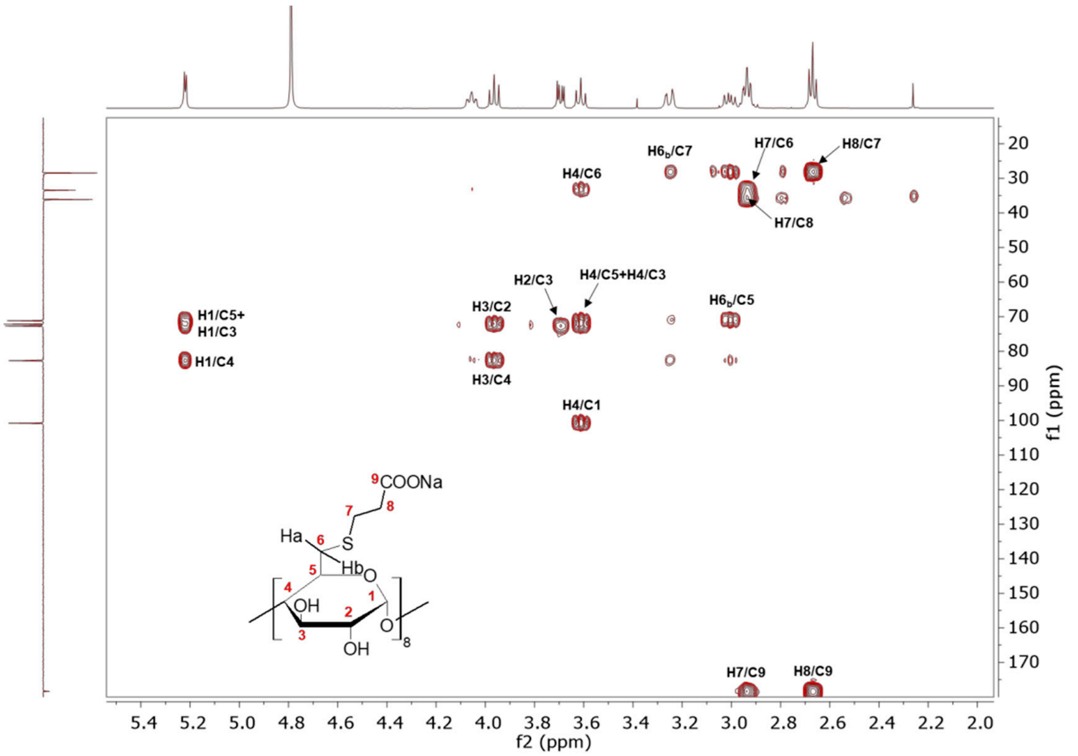

Figure S17. HMBC spectrum of Suγ-CD with full assignment (600 MHz, D_2O, 300 K).

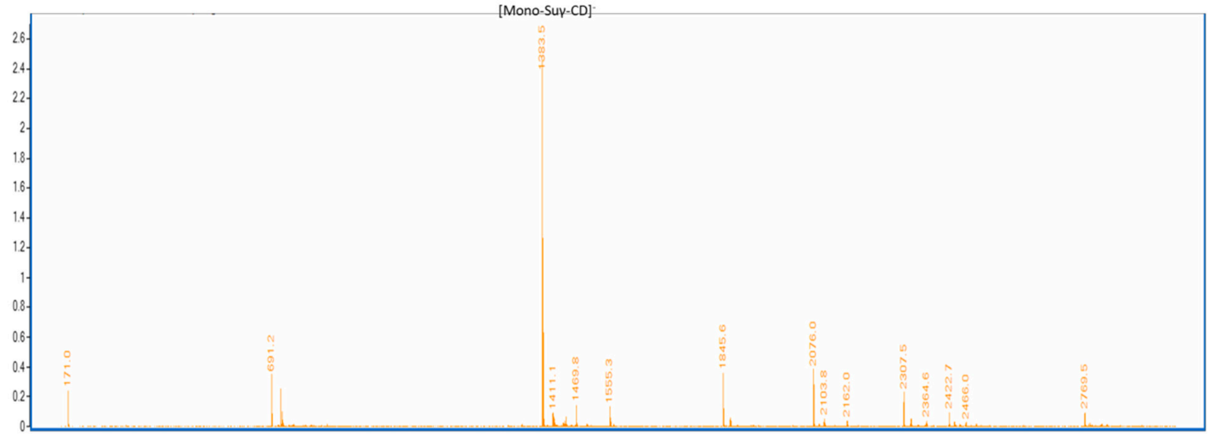

Figure S18. ESI-MS spectrum of Mono-Suγ-CD.

International Journal of *Molecular Sciences*

MDPI

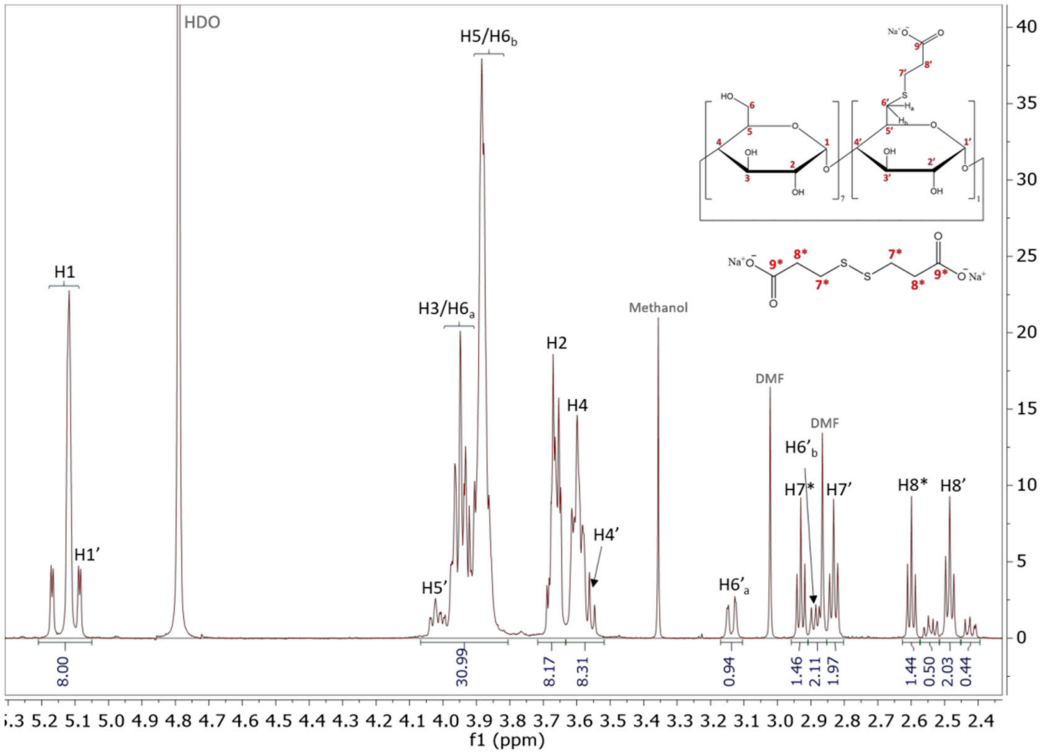

Figure S19. ^{1}H NMR spectrum of Mono-Suγ-CD with full assignment (600 MHz, D_2O, 300 K).

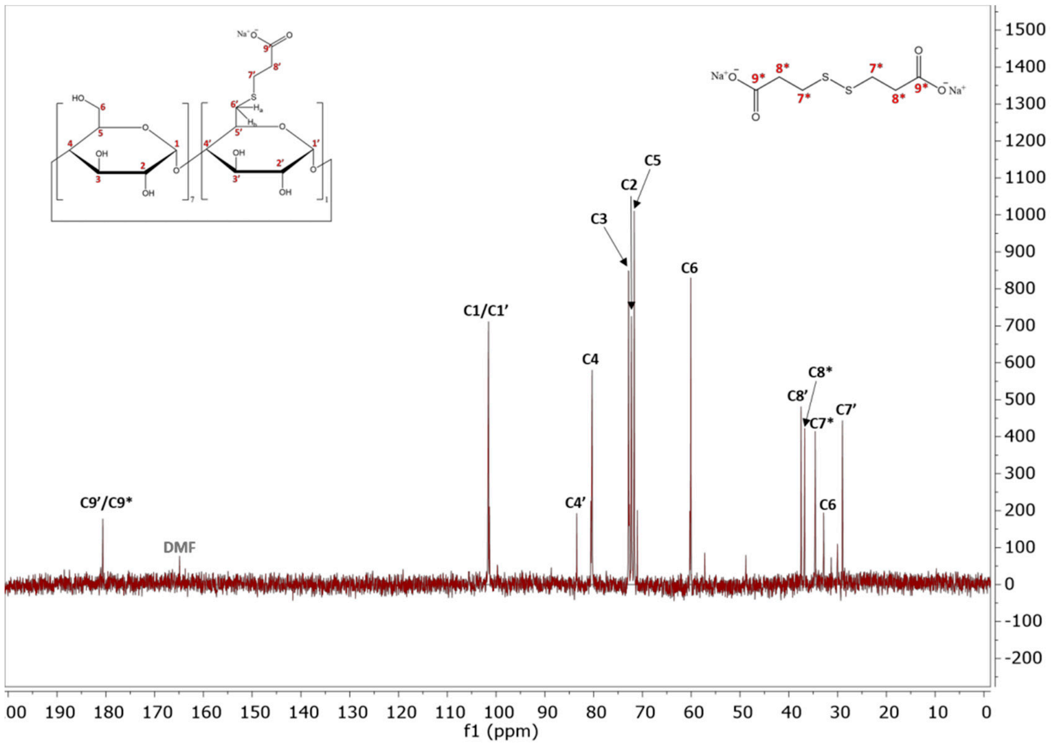

Figure S20. ^{13}C NMR spectrum of Mono-Suγ-CD with full assignment (150 MHz, D_2O, 300 K).

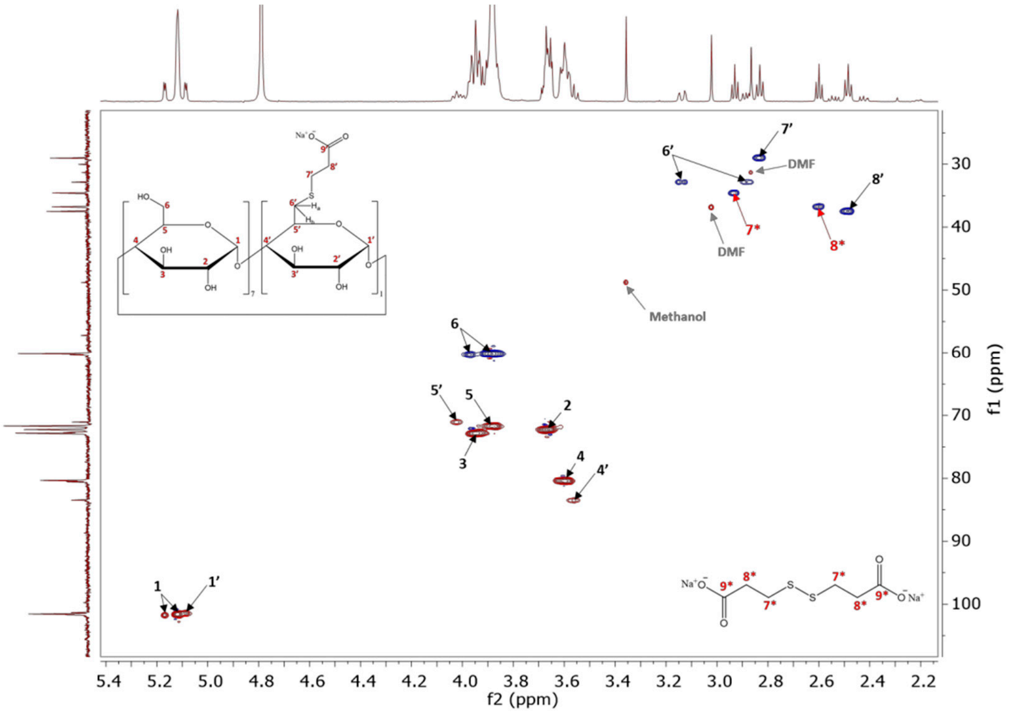

Figure S21. Multiplicity-edited HSQC spectrum of Mono-Suγ-CD with full assignment (600 MHz, D_2O, 300 K).

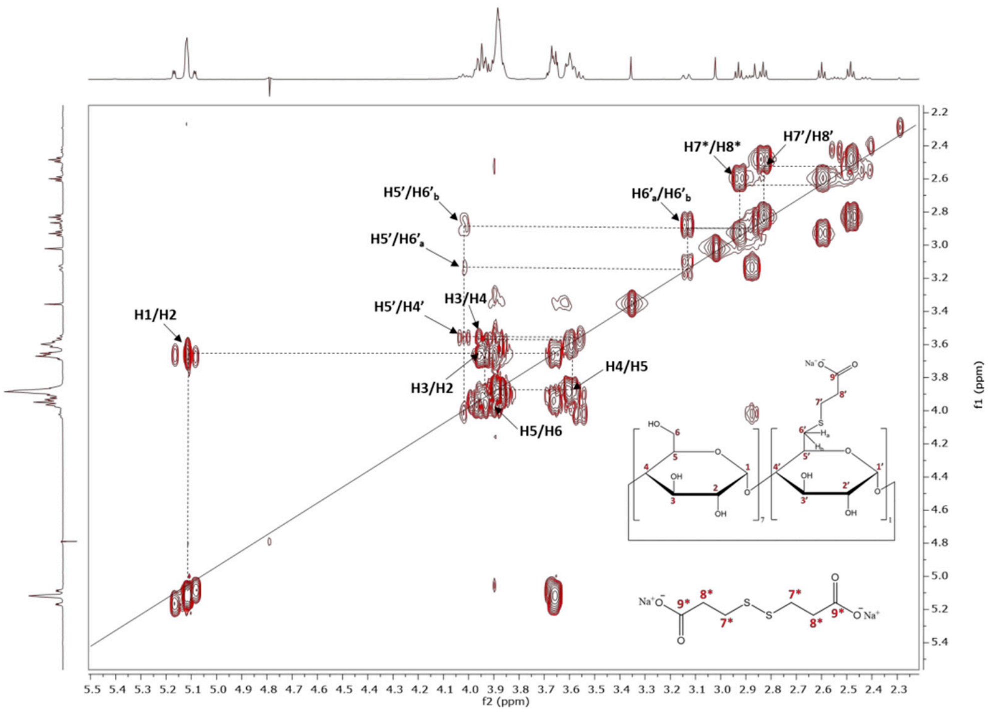

Figure S22. ^1H-^1H COSY spectrum of Mono-Suγ-CD with full assignment (600 MHz, D_2O, 300 K).

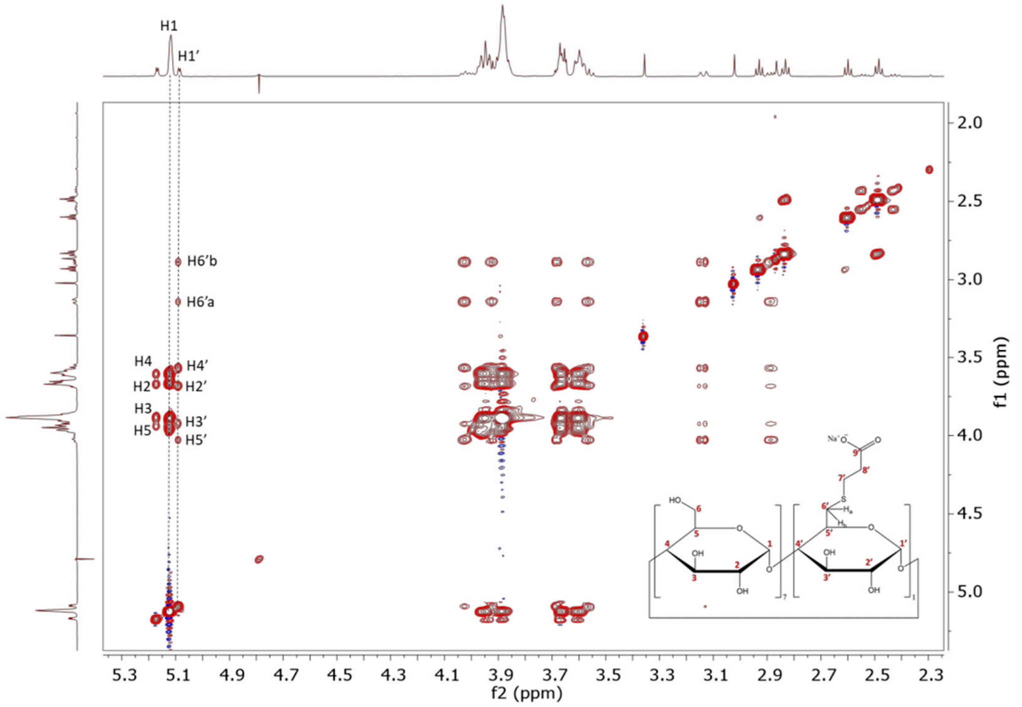

Figure S23. ^1H-^1H TOCSY spectrum of Mono-Suγ-CD with partial assignment (600 MHz, D_2O, 300 K).

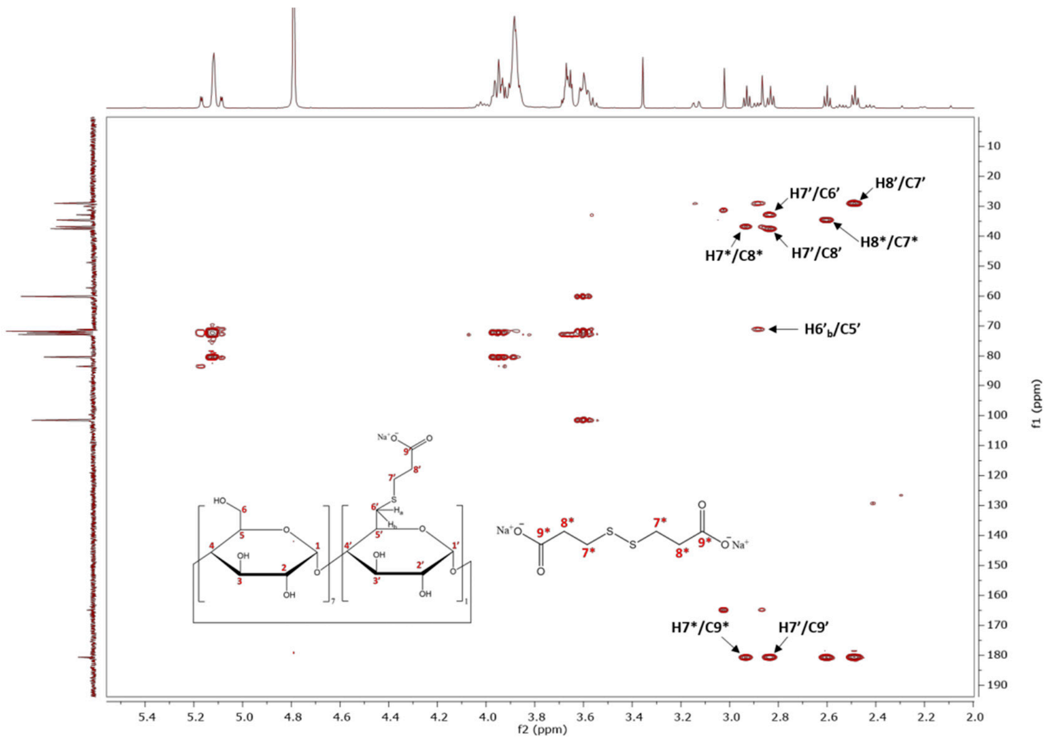

Figure S24. HMBC spectrum of Mono-Suγ-CD with partial assignment (600 MHz, D_2O, 300 K).

International Journal of
Molecular Sciences

MDPI

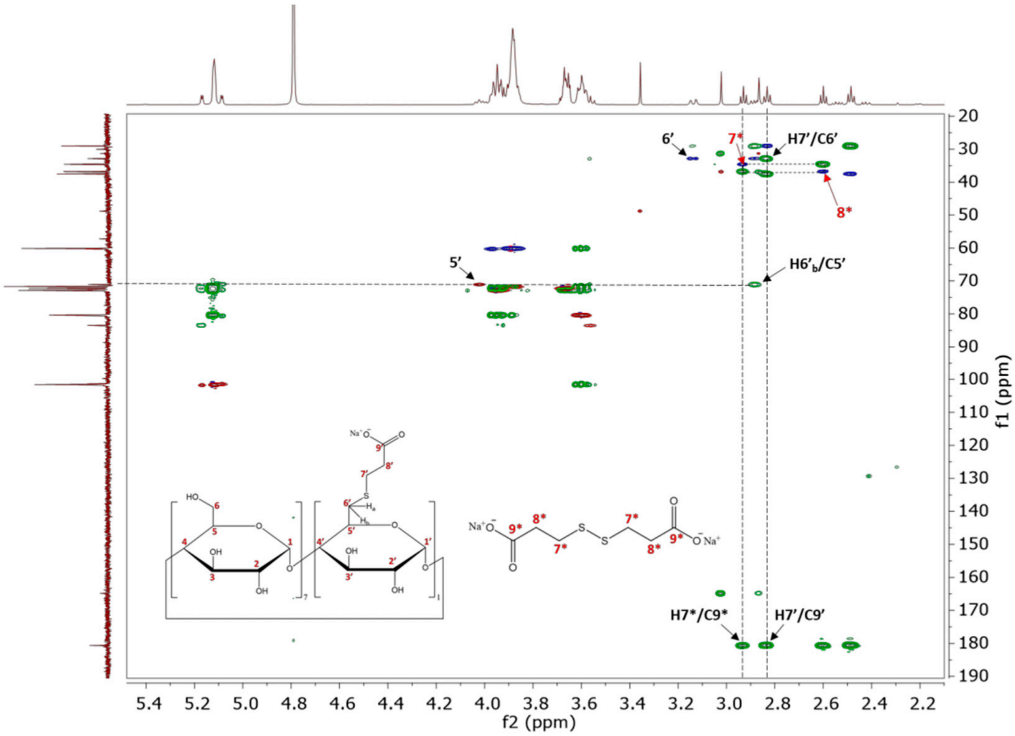

Figure S25. Superimposed multiplicity-edited HSQC- and HMBC spectra of Mono-Suγ-CD with partial assignment (600 MHz, D_2O, 300 K).

Ch2. The reduced microscopic protonation scheme of Sualphadex

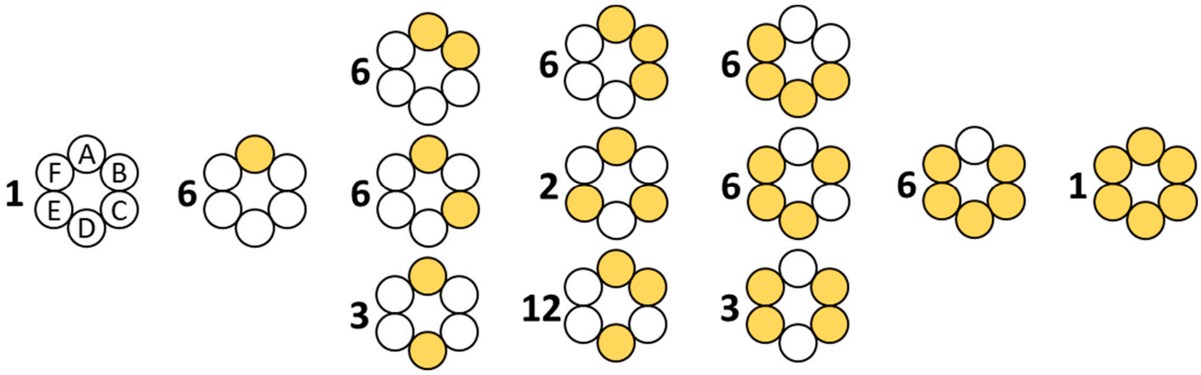

Figure S26. The reduced microscopic protonation scheme of Suα-CD, with multiplicities of the protonation isomers.

International Journal of Molecular Sciences

MDPI

Ch3. Detailed results of titration curve evaluations by R scripts

Table S1. Optimized basicity parameters and their statistical characteristics of Suα-CD calculated using the equidistant macroscopic model.

**Parameters**	**Estimate**	**Standard error**	**t value**	**Pr(>|t|)**
log K_1	6.4281035	0.0688476	93.37	<2e-16 ***
log K_2	5.7863927	0.0870236	66.49	<2e-16 ***
log K_3	5.2568017	0.1159593	45.33	<2e-16 ***
log K_4	4.6996079	0.1418424	33.13	<2e-16 ***
log K_5	4.5314771	0.1143074	39.64	<2e-16 ***
log K_6	3.7675316	0.0731355	51.51	<2e-16 ***
δ(L)	2.4637835	0.0007960	3095.14	<2e-16 ***
δ(H_6L)	2.7064428	0.0008206	3298.12	<2e-16 ***

Table S2. Correlation matrix of the optimized basicity parameters of Suα-CD calculated using the equidistant macroscopic model.

	**log K_1**	**log K_2**	**log K_3**	**log K_4**	**log K_5**	**log K_6**	**δ(L)**
**log K_2**	-0.72						
**log K_3**	0.58	-0.87					
**log K_4**	-0.38	0.67	-0.87				
**log K_5**	0.30	-0.52	0.72	-0.93			
**log K_6**	-0.13	0.30	-0.40	0.62	-0.73		
**δ(L)**	0.65	-0.25	-0.28	0.14	-0.14	0.04	
**δ(H_6L)**	0.04	-0.14	0.13	-0.28	0.29	-0.67	-0.02

Table S3. Optimized basicity parameters and their statistical characteristics of Suα-CD calculated using the macroscopic Q-fitting model.

**Parameters**	**Estimate**	**Standard error**	**t value**	**Pr(>|t|)**
logQ1	5.649953	0.068848	82.06	<2e-16 ***
logQ2	11.737374	0.060454	194.15	<2e-16 ***
logQ3	17.170269	0.094249	182.18	<2e-16 ***
logQ4	21.994814	0.096720	227.41	<2e-16 ***
logQ5	26.623202	0.075799	351.23	<2e-16 ***
logQ6	30.469915	0.086367	352.80	<2e-16 ***
δ(L)	2.463783	0.000796	3095.14	<2e-16 ***
$\Delta\delta$	0.242659	0.001156	210.00	<2e-16 ***

International Journal of Molecular Sciences

Table S4. Correlation matrix of the optimized basicity parameters of Suα-CD calculated using the macroscopic Q-fitting model.

	**log Q_1**	**log Q_2**	**log Q_3**	**log Q_4**	**log Q_5**	**log Q_6**	**δ(L)**
**log Q_2**	0.10						
**log Q_3**	0.78	-0.08					
**log Q_4**	0.21	0.70	-0.10				
**log Q_5**	0.72	0.27	0.81	0.14			
**log Q_6**	0.52	0.48	0.46	0.64	0.60		
**δ(L)**	-0.65	-0.38	-0.59	-0.37	-0.68	-0.56	
**Δδ**	0.47	0.15	0.44	0.00	0.45	-0.03	-0.70

Table S5. Optimized basicity parameters and their statistical characteristics of Suα-CD calculated using the microscopic site-binding model without considering any pair-interactivity parameters.

**Parameters**	**Estimate**	**Standard error**	**t value**	**Pr(>|t|)**
Log*k*	5.049482	0.048861	103.34	<2e-16 ***
δ(L)	2.472745	0.003374	732.94	<2e-16 ***
Δδ	0.226236	0.004626	48.91	<2e-16 ***

Table S6. Correlation matrix of the optimized basicity parameters of Suα-CD calculated using the microscopic site-binding model without considering any pair-interactivity parameters.

	**log *k***	**δ(L)**
**δ(L)**	-0.43	
**Δδ**	-0.01	-0.67

Table S7. Optimized basicity parameters and their statistical characteristics of Suα-CD calculated using the microscopic site-binding model considering only the adjacent pair-interactivity parameter $p\varepsilon_{12}$.

**Parameters**	**Estimate**	**Standard error**	**t value**	**Pr(>|t|)**
log *k*	5.5067350	0.0244718	225.02	<2e-16 ***
$p\varepsilon_{12}$	0.4446380	0.0199580	22.28	4.29e-16 ***
δ(L)	2.4650424	0.0009172	2687.72	<2e-16 ***
Δδ	0.2411547	0.0013347	180.69	<2e-16 ***

Table S8. Correlation matrix of the optimized basicity parameters of Suα-CD calculated using the microscopic site-binding model considering only the adjacent pair-interactivity parameter $p\varepsilon_{12}$.

	**log *k***	**$p\varepsilon_{12}$**	**δ(L)**
**$p\varepsilon_{12}$**	0.81		
**δ(L)**	-0.60	-0.39	
**Δδ**	0.44	0.56	-0.71

International Journal of Molecular Sciences

MDPI

Table S9. Optimized basicity parameters and their statistical characteristics of Suβ-CD calculated using the macroscopic Q-fitting model.

Parameters	Estimate	Standard error	t value	Pr(>|t|)
log Q_1	5.752e+00	3.244e-02	177.3	<2e-16 ***
log Q_2	1.224e+01	2.384e-02	513.4	<2e-16 ***
log Q_3	1.768e+01	7.092e-02	249.3	<2e-16 ***
log Q_4	2.333e+01	3.088e-02	755.4	<2e-16 ***
log Q_5	2.796e+01	5.128e-02	545.3	<2e-16 ***
log Q_6	3.250e+01	3.053e-02	1064.7	<2e-16 ***
log Q_7	3.626e+01	3.935e-02	921.3	<2e-16 ***
δ(L)	2.462e+00	2.849e-04	8639.5	<2e-16 ***
$\Delta\delta$	2.310e-01	3.919e-04	589.4	<2e-16 ***

Table S10. Correlation matrix of the optimized basicity parameters of Suβ-CD calculated using the macroscopic Q-fitting model.

	**log Q_1**	**log Q_2**	**log Q_3**	**log Q_4**	**log Q_5**	**log Q_6**	**log Q_7**	**δ(L)**
**log Q_2**	0.00							
**log Q_3**	0.74	-0.35						
**log Q_4**	0.28	0.72	-0.19					
**log Q_5**	0.62	-0.02	0.79	-0.10				
**log Q_6**	0.61	0.47	0.35	0.73	0.34			
**log Q_7**	0.60	0.27	0.56	0.37	0.75	0.65		
**δ(L)**	-0.66	-0.36	-0.48	-0.49	-0.53	-0.70	-0.61	
**$\Delta\delta$**	0.44	0.26	0.23	0.37	0.12	0.43	0.01	-0.71

Table S11. Optimized basicity parameters and their statistical characteristics of Suβ-CD calculated using the equidistant macroscopic model.

Parameters	Estimate	Standard error	t value	Pr(>|t|)
log K_1	6.5971796	0.0324428	203.35	<2e-16 ***
log K_2	6.1831127	0.0403412	153.27	<2e-16 ***
log K_3	5.2652963	0.0823162	63.96	<2e-16 ***
log K_4	5.5265209	0.0824662	67.02	<2e-16 ***
log K_5	4.5359851	0.0623279	72.78	<2e-16 ***
log K_6	4.4624095	0.0500880	89.09	<2e-16 ***
log K_7	3.6856807	0.0302087	122.01	<2e-16 ***
δ(L)	2.4616919	0.0002849	8639.54	<2e-16 ***
δ(H_7L)	2.6926937	0.0002762	9748.70	<2e-16 ***

International Journal of Molecular Sciences

Table S12. Correlation matrix of the optimized basicity parameters of Suβ-CD calculated using the equidistant macroscopic model.

	**log K_1**	**log K_2**	**log K_3**	**log K_4**	**log K_5**	**log K_6**	**log K_7**	**δ(L)**
**log K_2**	-0.81							
**log K_3**	0.64	-0.86						
**log K_4**	-0.53	0.77	-0.97					
**log K_5**	0.37	-0.52	0.75	-0.85				
**log K_6**	-0.27	0.39	-0.60	0.71	-0.94			
**log K_7**	0.17	-0.20	0.35	-0.41	0.65	-0.74		
**δ(L)**	-0.66	0.32	-0.31	0.23	-0.19	0.11	-0.09	
**$\delta(H_7L)$**	-0.06	0.05	-0.14	0.15	-0.31	0.31	-0.69	0.02

Table S13. Optimized basicity parameters and their statistical characteristics of Suβ-CD calculated using the microscopic site-binding model without considering any pair-interactivity parameters.

**Parameters**	**Estimate**	**Standard error**	**t value**	**Pr(>|t|)**
log k	5.169597	0.054987	94.02	<2e-16 ***
δ(L)	2.471192	0.003659	675.33	<2e-16 ***
$\Delta\delta$	0.212791	0.004916	43.29	<2e-16 ***

Table S14. Correlation matrix of the optimized basicity parameters of Suβ-CD calculated using the microscopic site-binding model without considering any pair-interactivity parameters.

	**log k**	**δ(L)**
**δ(L)**	-0.43	
**$\Delta\delta$**	0.01	-0.69

Table S15. Optimized basicity parameters and their statistical characteristics of Suβ-CD calculated using the microscopic site-binding model considering only the adjacent pair-interactivity parameter $p\varepsilon_{12}$.

**Parameters**	**Estimate**	**Standard error**	**t value**	**Pr(>|t|)**
log k	5.6969870	0.0148774	382.93	<2e-16 ***
$p\varepsilon_{12}$	0.5143729	0.0116815	44.03	<2e-16 ***
δ(L)	2.4623937	0.0005427	4537.10	<2e-16 ***
$\Delta\delta$	0.2295242	0.0007583	302.69	<2e-16 ***

Table S16. Correlation matrix of the optimized basicity parameters of Suβ-CD calculated using the microscopic site-binding model considering only the adjacent pair-interactivity parameter $p\varepsilon_{12}$.

	**log k**	**$p\varepsilon_{12}$**	**δ(L)**
**$p\varepsilon_{12}$**	0.80		
**δ(L)**	-0.64	-0.42	
**$\Delta\delta$**	0.47	0.57	-0.73

International Journal of Molecular Sciences

MDPI

Table S17. Optimized basicity parameters and their statistical characteristics of Suγ-CD calculated using the macroscopic Q-fitting model.

Parameters	Estimate	Standard error	t value	Pr(>|t|)
log Q_1	5.774e+00	7.499e-02	77.0	<2e-16 ***
log Q_2	1.225e+01	6.962e-02	175.9	<2e-16 ***
log Q_3	1.831e+01	1.115e-01	164.1	<2e-16 ***
log Q_4	2.371e+01	1.316e-01	180.1	<2e-16 ***
log Q_5	2.895e+01	1.169e-01	247.6	<2e-16 ***
log Q_6	3.362e+01	1.002e-01	335.6	<2e-16 ***
log Q_7	3.803e+01	7.452e-02	510.4	<2e-16 ***
log Q_8	4.187e+01	7.900e-02	530.1	<2e-16 ***
δ(L)	2.459e+00	5.522e-04	4452.6	<2e-16 ***
$\Delta\delta$	2.369e-01	7.453e-04	317.9	<2e-16 ***

Table S18. Correlation matrix of the optimized basicity parameters of Suγ-CD calculated using the macroscopic Q-fitting model.

	**log Q_1**	**log Q_2**	**log Q_3**	**log Q_4**	**log Q_5**	**log Q_6**	**log Q_7**	**log Q_8**	**δ(L)**
**log Q_2**	-0.35								
**log Q_3**	0.81	-0.60							
**log Q_4**	-0.12	0.73	-0.55						
**log Q_5**	0.66	-0.44	0.87	-0.63					
**log Q_6**	0.25	0.41	-0.05	0.72	-0.29				
**log Q_7**	0.72	-0.10	0.70	-0.13	0.77	0.07			
**log Q_8**	0.58	0.13	0.44	0.28	0.35	0.67	0.57		
**δ(L)**	-0.71	-0.05	-0.58	-0.12	-0.53	-0.39	-0.71	-0.63	
**$\Delta\delta$**	0.55	-0.05	0.48	-0.06	0.47	0.01	0.56	0.10	-0.76

Table S19. Optimized basicity parameters and their statistical characteristics of Suγ-CD calculated using the equidistant macroscopic model.

Parameters	Estimate	Standard error	t value	Pr(>|t|)
log K_1	6.6775505	0.0749883	89.05	<2e-16 ***
log K_2	6.1708023	0.1189614	51.87	<2e-16 ***
log K_3	5.8833359	0.1631004	36.07	5.43e-16 ***
log K_4	5.2807689	0.2146101	24.61	1.54e-13 ***
log K_5	5.1425885	0.2242294	22.93	4.30e-13 ***
log K_6	4.5919859	0.1743635	26.34	5.67e-14 ***
log K_7	4.3419606	0.1208483	35.93	5.76e-16 ***
log K_8	3.7831809	0.0709155	53.35	<2e-16 ***
δ(L)	2.4587918	0.0005522	4452.57	<2e-16 ***
δ(H_8L)	2.6957094	0.0004880	5523.85	<2e-16 ***

International Journal of Molecular Sciences

MDPI

Table S20. Correlation matrix of the optimized basicity parameters and their statistical characteristics of Suγ-CD calculated using the equidistant macroscopic model.

	**log K_1**	**log K_2**	**log K_3**	**log K_4**	**log K_5**	**log K_6**	**log K_7**	**log K_8**	**δ(L)**
**log K_2**	-0.84								
**log K_3**	0.70	-0.93							
**log K_4**	-0.50	0.76	-0.91						
**log K_5**	0.42	-0.65	0.81	-0.97					
**log K_6**	-0.30	0.49	-0.64	0.83	-0.92				
**log K_7**	0.23	-0.38	0.50	-0.66	0.77	-0.93			
**log K_8**	-0.11	0.22	-0.28	0.41	0.48	-0.67	-0.82		
**δ(L)**	-0.71	0.42	-0.37	0.22	-0.20	0.13	-0.11	0.04	
**δ(H_8L)**	0.04	-0.10	0.11	-0.19	0.20	-0.33	0.38	-0.68	-0.02

Table S21. Optimized basicity parameters and their statistical characteristics of Suγ-CD calculated using the microscopic site-binding model without considering any pair-interactivity parameters.

**Parameters**	**Estimate**	**Standard error**	**t value**	**Pr(>|t|)**
log *k*	5.219900	0.055369	94.28	<2e-16 ***
δ(L)	2.468329	0.003803	649.11	<2e-16 ***
Δδ	0.219463	0.005054	43.42	<2e-16 ***

Table S22. Correlation matrix of the optimized basicity parameters of Suγ-CD calculated using the microscopic site-binding model without considering any pair-interactivity parameters.

	**log *k***	**δ(L)**
**δ(L)**	-0.44	
**Δδ**	0.03	-0.69

Table S23. Optimized basicity parameters and their statistical characteristics of Suγ-CD calculated using the microscopic site-binding model considering only the adjacent pair-interactivity parameter $p\varepsilon_{12}$.

**Parameters**	**Estimate**	**Standard error**	**t value**	**Pr(>|t|)**
log *k*	5.7348507	0.0105889	541.6	<2e-16 ***
$p\varepsilon_{12}$	0.5029535	0.0083551	60.2	<2e-16 ***
δ(L)	2.4591174	0.0004057	6060.9	<2e-16 ***
Δδ	0.2364180	0.0005586	423.2	<2e-16 ***

Table S24. Correlation matrix of the optimized basicity parameters of Suγ-CD calculated using the microscopic site-binding model considering only the adjacent pair-interactivity parameter $p\varepsilon_{12}$.

	**log *k***	**$p\varepsilon_{12}$**	**δ(L)**
**$p\varepsilon_{12}$**	0.80		
**δ(L)**	-0.64	-0.42	
**Δδ**	0.48	0.56	-0.74

Ch4. Calculated macrospecies distributions and charge-pH profiles

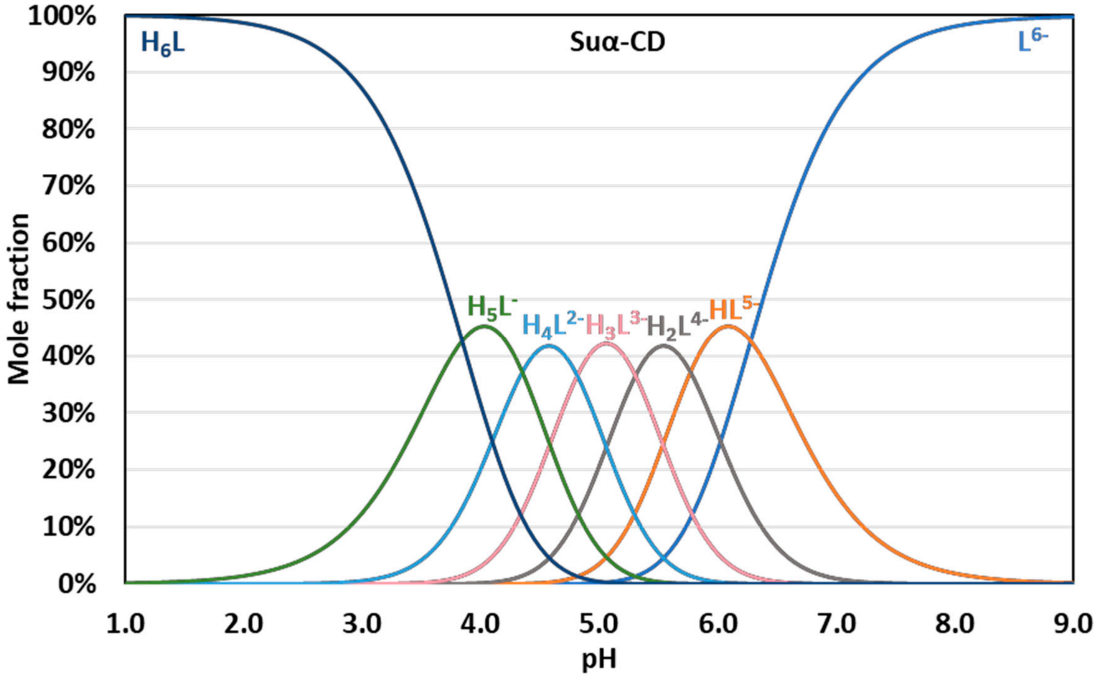

Figure S27. pH-dependent distribution of protonation microspecies of Suα-CD**.**

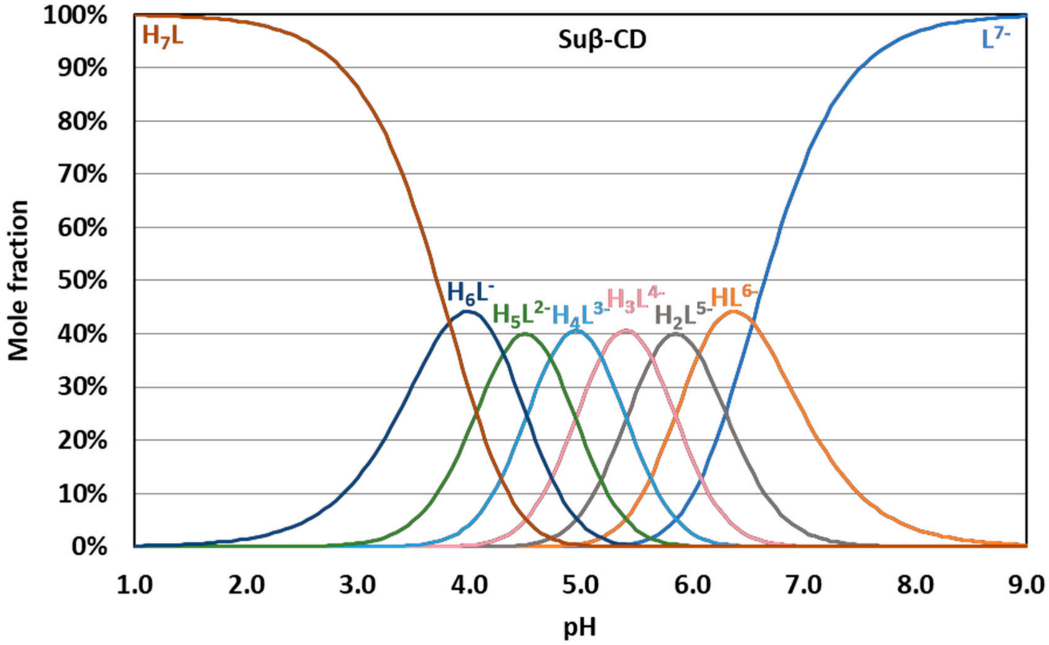

Figure S28. pH-dependent distribution of protonation microspecies of Suβ-CD.

International Journal of
Molecular Sciences

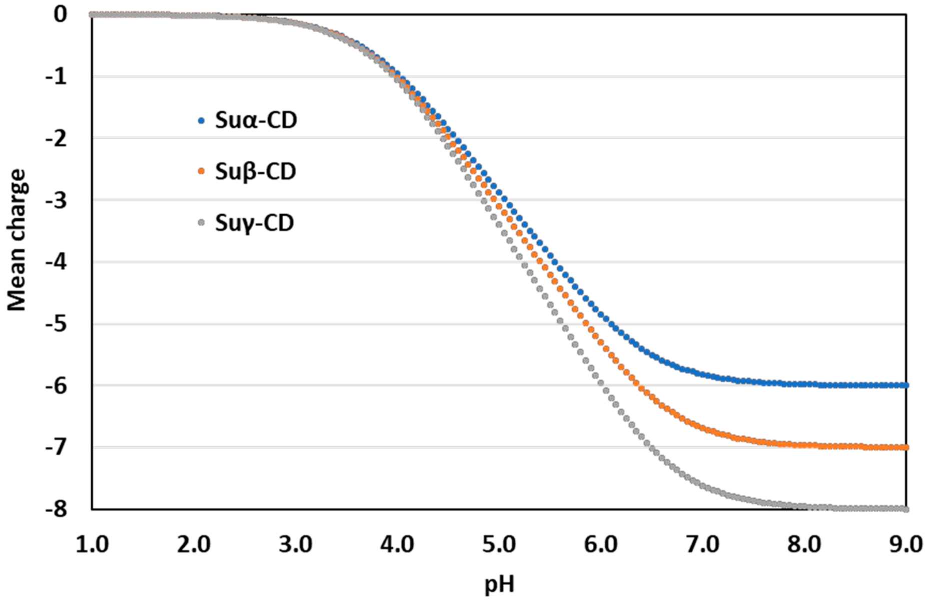

Figure S29. Mean charge of Suα-CD, Suβ-CD and Suγ-CD as functions of pH.

International Journal of
Molecular Sciences

MDPI

Ch5. Acid-base profiling of related compounds

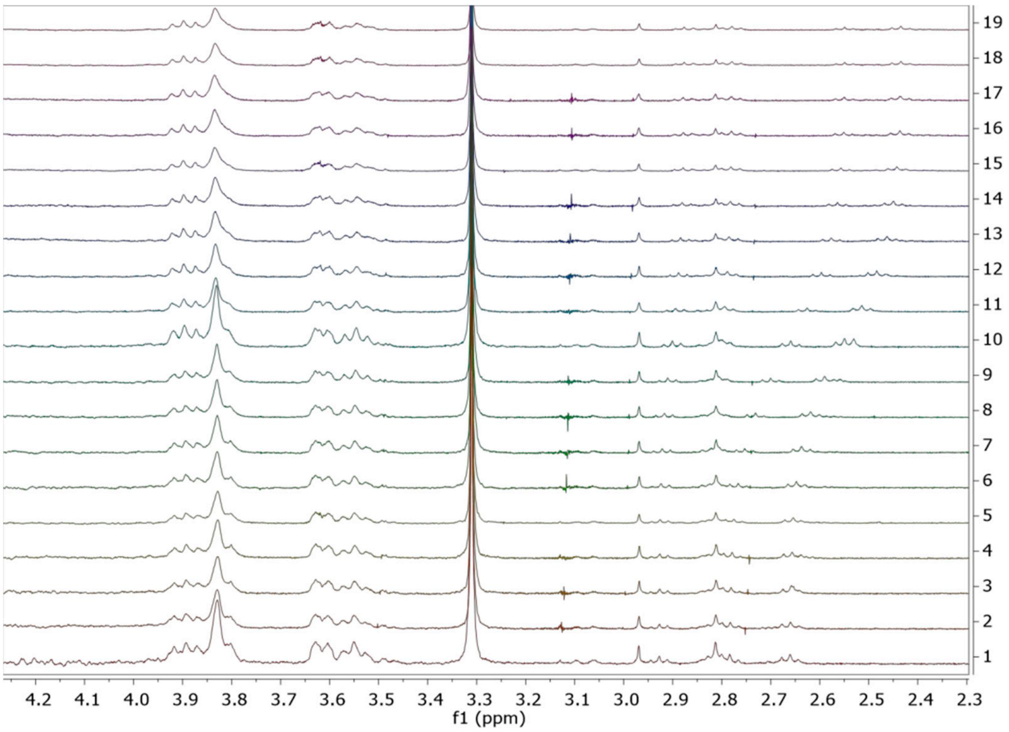

Figure S30. pH dependent ^{1}H NMR spectra of Mono-Suγ-CD (400 MHz, D_2O, 300 K).

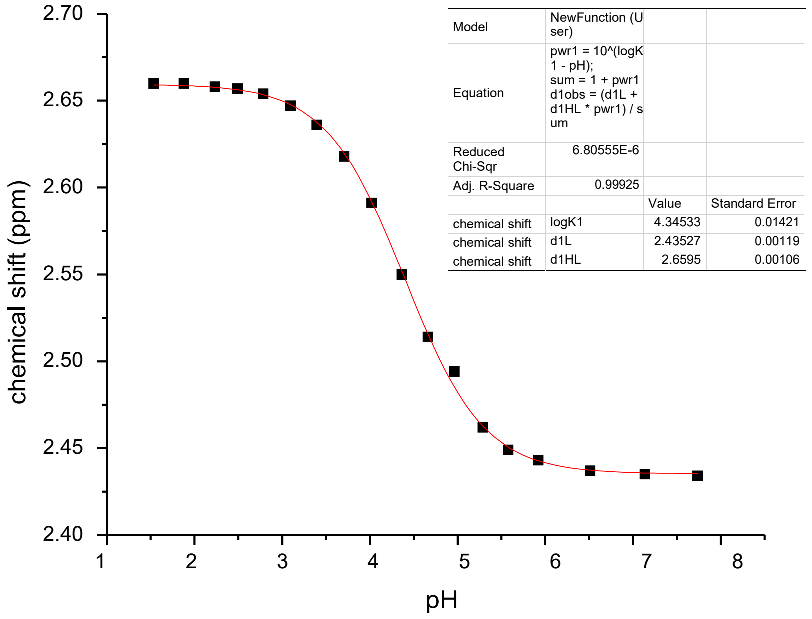

Figure S31. The experimental pH-dependent chemical shifts of H8 of Mono-Suγ-CD in squares, while red line represents the titration curves fitted (400 MHz, D_2O, 300 K).

International Journal of *Molecular Sciences*

MDPI

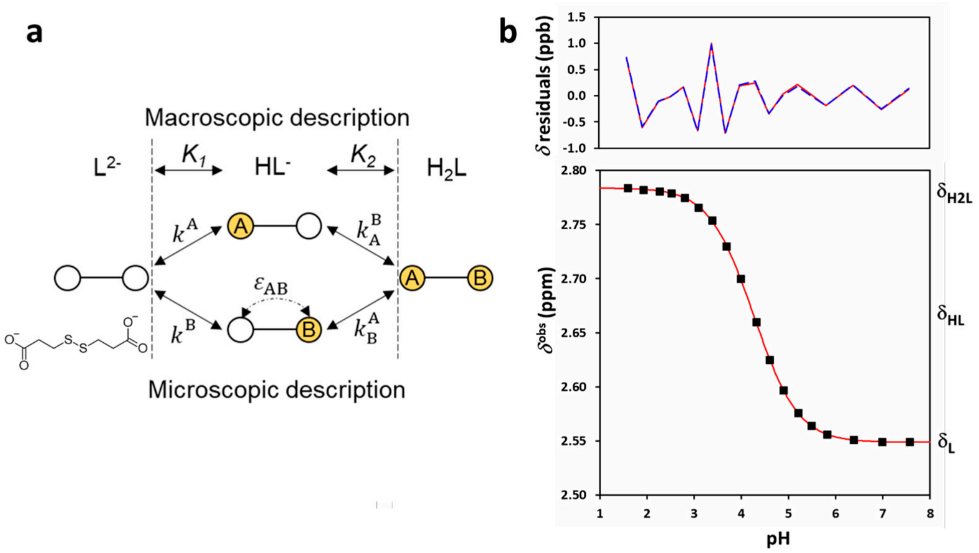

Figure S32. a) Macroscopic, microscopic protonation schemes and the molecular formula of the DTDPA dianion. Empty circles symbolize its equivalent A and B carboxylate sites in unprotonated, while filled circles in protonated state. b) The experimental pH-dependent chemical shifts of α-CH_2 in squares, while red line represents the computer-fitted curves. The residuals by fitting of the macroscopic and microscopic evaluations are given in red and blue lines above.

Table S25. Basicity parameters and ^{1}H NMR data of DTDPA (with standard deviations as error estimates in parenthesis) from three data evaluation approaches.

Evaluation	Macroscopic, Eq. (2)	Microscopic, Eq. (3)	ED macro, Eq. (7)	Literature**
log K_1	4.62 (0.06)	4.630 (0.005)*	4.631 (0.005)	4.47
log K_2	3.87 (0.07)	3.88 (0.01)*	3.883 (0.005)	3.88
log k^A	4.32 (0.06)*	4.330 (0.005)	4.330 (0.005)*	4.16
pε_{AB}	0.15 (0.01)*	0.15 (0.01)	0.15 (0.01)*	-0.01
δ_L	2.5487 (0.0003)	2.5487 (0.0002)	2.5487 (0.0002)	-
δ_{HL}	2.67 (0.02)	2.6663 (0.0002)*	2.6663 (0.0002)*	-
δ_{H_2L}	2.7839 (0.0003)	2.7839 (0.0002)*	2.7839 (0.0002)	-
$\Delta\delta$	0.2351 (0.0004)*	0.2351 (0.0004)	0.2351 (0.0004)*	-

*calculated by the quadratic error propagation law including the respective correlation coefficient. The realistic precision is two decimals for the logarithmic basicity parameters and three decimals for the chemical shifts in ppm. Extra digits are given for the sake of numerical comparison of the evaluation approaches

** C.J. Hawkins, D.D. Perrin, Polynuclear Complex Formation. II. Copper(II) with Cystine and Related Ligands, Inorg. Chem. 2 (1963) 843–849. https://doi.org/10.1021/ic50008a043.
